# Nanocomposites of Poly(*n*-Butyl Acrylate) with Fe_3_O_4_: Crosslinking with Hindered Urea Bonds, Reprocessing and Related Functional Properties

**DOI:** 10.3390/polym16182638

**Published:** 2024-09-18

**Authors:** Lei Li, Huaming Wang, Xibin Shen, Guohua Hang, Yuan Gao, Jiawei Hu, Sixun Zheng

**Affiliations:** Department of Polymer Science and Engineering and the State Key Laboratory of Metal Matrix Composites, Shanghai Jiao Tong University, Shanghai 200240, China

**Keywords:** nanocomposites, hindered urea bonds, Fe_3_O_4_, photo- and magnetic–thermal conversion, reprocessing, shape memory

## Abstract

In this contribution, we reported the synthesis of the nanocomposites of poly(*n*-butyl acrylate) with Fe_3_O_4_ nanoparticles (NPs) via dynamic crosslinking of poly(*n*-butyl acrylate)-grafted Fe_3_O_4_ NPs with hindered urea bonds (HUBs). Towards this end, the surfaces of Fe_3_O_4_ NPs were grafted with poly(*n*-butyl acrylate-*ran*-2-(3-*tert*-butyl-3-ethylureido)ethyl acrylate) chains [denoted as Fe_3_O_4_-*g*-P(BA-*r*-TBEA)] via living radical polymerization. Thereafter, 1,2-bis(tert-butyl)ethylenediamine was used as a crosslinker to afford the organic–inorganic networks with variable contents of Fe_3_O_4_ NPs and crosslinking densities. It was found that the fine dispersion of Fe_3_O_4_ NPs in the matrix of poly(*n*-butyl acrylate) was achieved. The nanocomposites exhibited excellent reprocessing properties, attributed to the introduction of HUBs. Owing to the crosslinking, the nanocomposites displayed excellent shape memory properties. Further, the nanocomposites possessed photo- and magnetic–thermal properties, which were inherited from Fe_3_O_4_ NPs. These functional properties allow triggering the shape shifting of the nanocomposites in an uncontacted fashion.

## 1. Introduction

Organic–inorganic composites have gained tremendous attention in various technological fields [1,2,3]. By combining appropriate building blocks with specific morphologies and functionalities, the composites with tailored multiple properties can be developed. Magnetic nanoparticles (NPs), e.g., superparamagnetic Fe_3_O_4_ NPs, hold great potential for the use in biomedical applications and hybrid designs [4,5]. Substantive amounts of research work have been reported that Fe_3_O_4_ NPs serve as essential components for the generation of nanocomposites with comprehensive performances [6,7,8]. It has been realized that the uniform dispersion of Fe_3_O_4_ NPs in the polymer matrix is crucial for optimizing the properties of the composites. However, this task is challenging because Fe_3_O_4_ NPs tend to aggregate in the polymer matrix due to their strong magnetic interactions and their inherent incompatibility with organic polymers [9]. Surface functionalization of Fe_3_O_4_ NPs has been demonstrated to be one of the most effective approaches to achieve uniform dispersion. Grafting polymers is one of the most effective methods since this approach allows significant alterations in surface properties by selecting various functional entities. Surface functionalization of Fe_3_O_4_ NPs can be achieved with two approaches: (i) “grafting to” and (ii) “grafting from”. The “grafting to” method involves the surface reaction of the polymers with reactive end groups [10]. Notably, the presence of initially attached polymers may restrict the addition of other polymers, limiting density and coverage [11]. In contrast, “grafting from” entails surface-initiatedpolymerizations, which generally leads to higher graft densities due to the much higher diffusion rates of monomers than polymers. In recent years, surface-initiated radical polymerizations, such as atom transfer radical polymerization (ATRP) and reversible addition–fragmentation chain transfer (RAFT) polymerization, have been widely reported [12,13,14,15,16]. With these approaches, compositions and morphologies of Fe_3_O_4_–polymer nanocomposites can be well controlled.

Incorporating dynamic covalent bonds into crosslinked polymer networks provides an effective approach to impart malleability and processability to polymeric materials. This is achieved through network rearrangements driven by the exchange reaction of dynamic covalent bonds in response to external stimuli like heat, light or magnetic fields [17,18,19,20]. The development of dynamic crosslinked networks allows for the thermal processing of crosslinked polymer networks in a manner similar to processing of thermoplastic polymers, offering an effective approach for recycling thermosets [21,22,23]. Additionally, the introduction of dynamic covalent bonds significantly alters the production of crosslinked polymer networks, enabling post-processing even after crosslinking. Leibler et al. [24] first reported the utilization of this approach. In their work, transesterification was used as the dynamic chemistry on the basis of hydroxyl ether ester structural units. In the presence of a catalyst, hydroxyl ether ester structural units behaved as dynamic covalent bonds, transesterification of which allows re-curation of epoxy networks. As a result, epoxy networks were reprocessable at elevated temperatures. Since then, various dynamic covalent bonds, including disulfide [25,26,27], imines [28,29,30], olefin metathesis [31,32,33], boronic ester bonds [34,35,36], transesterification [37,38,39], vinylogous urethane bonds [40,41,42] and hindered urea bonds (HUBs), have been employed to impart reprocessing or self-healing properties to crosslinked polymers. Among them, HUBs are notable for their role in fabricating dynamic crosslinking networks, formed by the reaction of hindered amines with isocyanates. HUBs readily break and reform under mild conditions, allowing the reprocessing or self-healing of crosslinked polymers. Cheng et al. [43], for the first time, reported the introduction of HUBs into crosslinked poly(urethane-urea) (PUU) networks. These networks exhibited self-healing properties that varied with type of HUBs. In recent years, HUBs have been also introduced into non-PUU networks [44,45,46,47,48,49,50]. For example, Zhou et al. [44] reported polyurea/polydopamine (DCPU/PDA) nanocomposites using hindered urea bonds and polydopamine (PDA) particles as the dynamic motifs and functional nanofillers, respectively. These nanocomposites displayed fast responsive self-healing capabilities with the exchange of the HUBs and the effective photothermal properties of PDA particles. More recently, Zheng et al. [45] synthesized poly(*n*-butyl acrylate)-POSS linear copolymers carrying HUBs. Notably, a facile and efficient approach was developed to crosslink the linear copolymers via dynamic urea exchange reaction in the presence of difunctional hindered amine [viz. 1,2-bis(*tert*-butyl)ethylenediamine (BBED)]. The resulting crosslinked networks exhibited excellent reprocessing and self-healing properties. To date, there have been no reports of Fe_3_O_4_-based dynamic crosslinked networks involving HUBs.

In this work, we synthesized poly(*n*-butyl acrylate-*r*-[2-(3-*tert*-butyl-3-ethylureido)ethyl acrylate]-grafted Fe_3_O_4_ hybrid copolymers [denoted as Fe_3_O_4_-*g*-P(BA-*r*-TBEA)] bearing mono-HUBs via surface-initiated RAFT polymerization, in which the surface of Fe_3_O_4_ nanoparticles were functionalized with trithiocarbonate moieties and then served as the chain transfer agents, and the statistical copolymer chains of BA with TBEA were grown from the surfaces of the inorganic nanoparticles [51,52]. The Fe_3_O_4_-*g*-P(BA-*r*-TBEA) samples with various contents of mono-HUBs were subsequently crosslinked through a dynamic urea exchange reaction with BBED. Thanks to the dynamic urea exchange, the organic–inorganic nanocomposites were obtained, which further enabled the reprocessing of materials. Furthermore, due to the introduction of Fe_3_O_4_ NPs, the nanocomposites exhibited multi-stimuli-responsive (e.g., thermal, near-infrared NIR, magnetic) shape memory properties. In the meantime, the original shapes of the materials can be reprogrammed.

## 2. Experimental

### 2.1. Materials

**Scheme 1:**
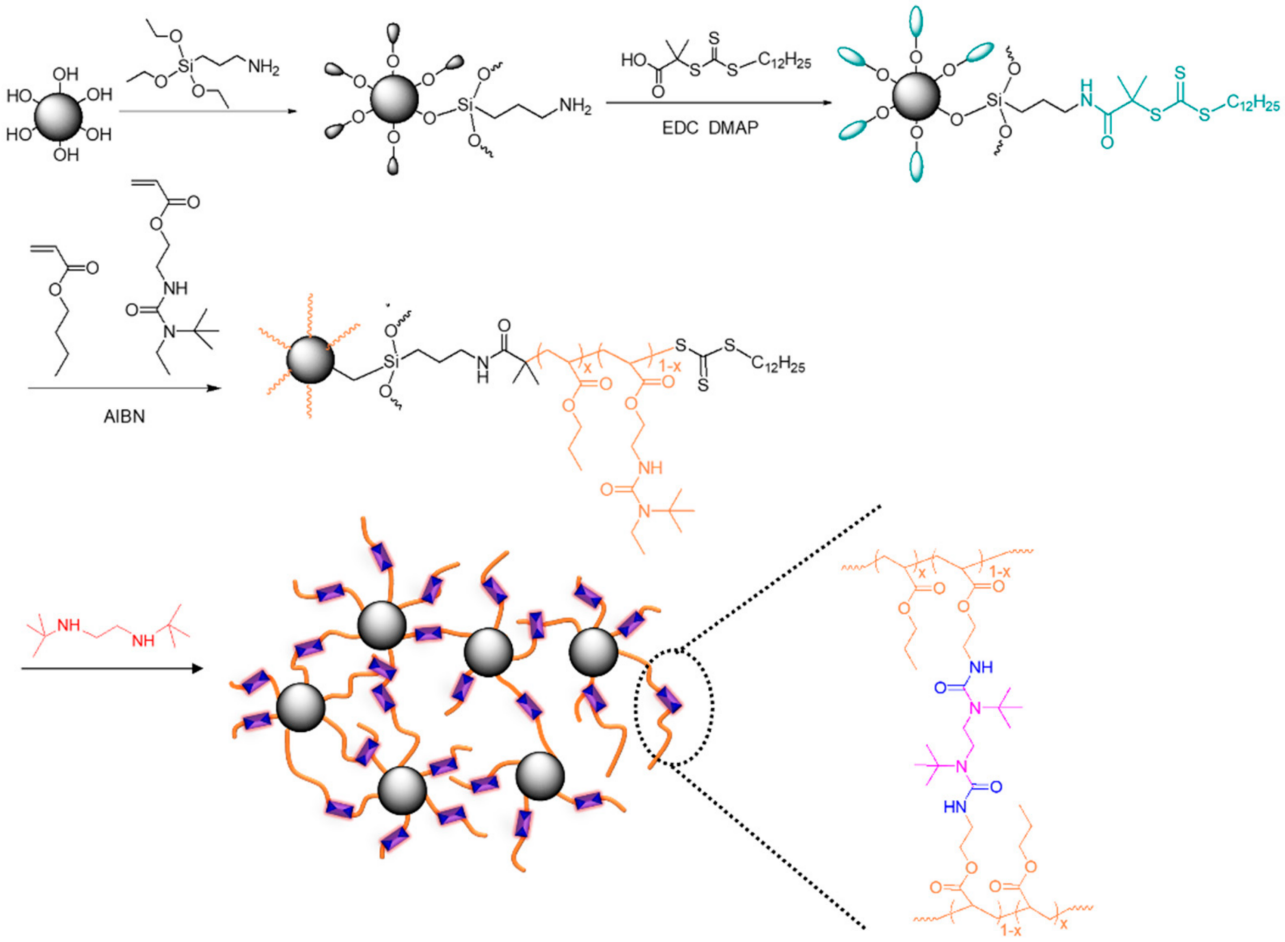
The synthesis of Fe_3_O_4_-*g*-P(BA-*r*-TBEA) nanocomposites.

*n*-Butyl acrylate (BA) was purchased from Shanghai Titan Scientific Co, Ltd., Shanghai, China. 2,2′-Azobis(2-methylpropionitrile) (AIBN), 2-isocyanatoethyl acrylate and 1,2-bis(*tert*-butyl)ethylenediamine (BBED) were purchased from TCI Co., Shanghai, China. 2-(3-*tert*-Butyl-3-ethylureido)ethylacrylate (TBEA) was synthesized by following the approach of literature [53], and its ^1^H NMR spectrum is presented in Figure S1. Pristine Fe_3_O_4_ nanoparticles (denoted Fe_3_O_4_-OH), aminopropyl-functionalized Fe_3_O_4_ nanoparticles (denoted Fe_3_O_4_-NH_2_) and trithiocarbonate-functionalized Fe_3_O_4_ nanoparticles (denoted Fe_3_O_4_-CTA) were synthesized by following the approach of literature [16], as detailed in Scheme 1. S-1-Dodecyl-s′-(α,α′-dimethyl-α″-acetic acid) trithiocarbonate (DDMAT) was used as the chain transfer agent for the RAFT polymerization; it was synthesized as reported by McCormick et al. [54]. The organic solvents such as 1,4-dioxane and petroleum ether were purchased from Shanghai Titan Scientific Co, Ltd., China.

### 2.2. Synthesis of Fe_3_O_4_-g-P(BA-r-TBEA)

Typically, Fe_3_O_4_-CTA (1.000 g), BA (2.500 g, 19.5 mmol), 2-(3-*tert*-Butyl-3-ethylureido)ethylacrylate (TBEA) (1.500 g, 6.19 mmol), AIBN (0.010 g, 0.03 mmol) and 1,4-dioxane (5 mL) were added to a flask. The mixture was bubbled with highly pure nitrogen for 30 min and then the polymerization was conducted at 70 °C for 24 h. Cooled to room temperature, the reacted mixture was dropwise added into 50 mL of petroleum ether to obtain the precipitates. After drying under vacuum, the product (4.945 g) was obtained, with a monomer conversion of 98.9%. By adjusting the mass ratios of Fe_3_O_4_-CTA to the monomers (i.e., BA + TBEA), Fe_3_O_4_-*g*-P(BA-*r*-TBEA)s were synthesized with (i) a fixed content of Fe_3_O_4_-CTA (viz. 15 wt%) and variable contents of TBEA and (ii) a fixed content of TBEA (viz. 20 wt%) and variable contents of Fe_3_O_4_. The products were named as Fe_3_O_4_a-*g*-P(BAb-*r*-TBEAc), where a, b and c represented the mass percentage of Fe_3_O_4_-CTA, BA and TBEA, respectively. The compositions of the products are summarized in Table S1.

### 2.3. Crosslinking of Fe_3_O_4_-g-P(BA-r-TBEA)

Typically, Fe_3_O_4_20-*g*-P(BA50-*r*-TBEA30) (3.000 g, 3.72 mmol with regard to HUBs) and 1,4-dioxane (5 mL) were added to a flask. 1,2-bis(*tert*-Butyl)ethylenediamine (BBED) (0.318 g, 1.85 mmol) was then added and the mixture was heated to 90 °C. With the proceeding of the reaction, the mixture was gradually gelled. To ensure the reaction was conducted completely, the low-molecular-weight product (viz. *tert*-butylethylamine) was distilled out. The dynamic crosslinking reaction was performed at 90 °C for 12 h. Subsequently, the gelled product was dried until a constant weight was obtained. The final crosslinked network weighed 2.950 g, resulting in a yield of 98.3%.

## 3. Results and Discussion

### 3.1. Synthesis of Fe_3_O_4_-g-P(BA-r-TBEA)

Poly(*n*-butyl acrylate-*r*-2-(3-*tert*-butyl-3-ethylureido)ethylacrylate)-grafted Fe_3_O_4_ nanoparticles [denoted Fe_3_O_4_-*g*-P(BA-*r*-TBEA)] were synthesized via surface-initiated RAFT polymerization, as shown in Scheme 1. Towards this end, pristine Fe_3_O_4_ NPs were surface-functionalized with trithiocarbonate moieties and the surface-functionalized Fe_3_O_4_ NPs were used to mediate the radical polymerization. To achieve the functionalization, pristine Fe_3_O_4_ NPs (i.e., Fe_3_O_4_-OH) were first treated with aminopropyl groups via the reaction of Fe_3_O_4_-OH with 3-aminopropyltriethoxysilane (APTES); the as-obtained surface aminopropyl groups were then allowed to react with *S*-1-dodecyl-s′-(α,α′-dimethyl-α″-acetic acid)trithiocarbonate (DDMAT) to obtain the Fe_3_O_4_ NPs bearing trithiocarbonate moieties (i.e., Fe_3_O_4_-CTA). Through FTIR spectroscopy, the surface functionalization of Fe_3_O_4_ NPs was investigated (Figure S2). To determine the portion of surface organic components, these nanoparticles were subjected to thermal gravimetric analysis (TGA). The TGA measurements were performed under air atmosphere. In this case, all of the organic components were decomposed at elevated temperature (e.g., 800 °C) whereas the inorganic portion (i.e., Fe_3_O_4_) remained almost invariant (99.1%). Therefore, the yields of degradation were utilized to estimate the grafting quantity of organic components at the surfaces of Fe_3_O_4_ NPs, as detailed in Supporting Information (SI) (Figure S3). In this work, the portion of trithiocarbonate moieties at the surfaces of Fe_3_O_4_ NPs was calculated to be 0.345 mmol × g^−1^.

**Figure 1:**
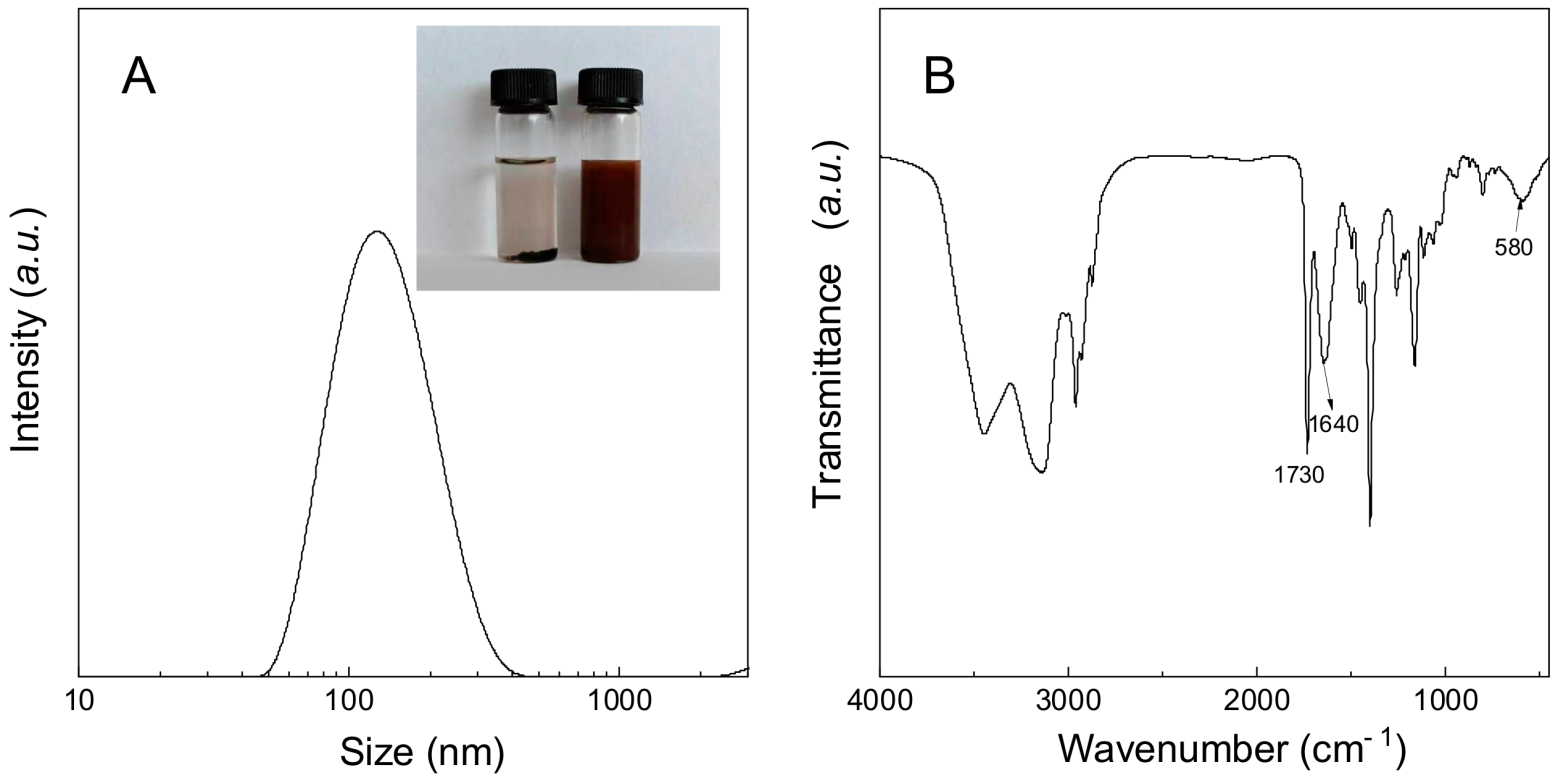
(**A**) DLS curve of Fe_3_O_4_15-*g*-P(BA65-*r*-TBEA20) in 1,4-dioxide at the concentration of 0.2 g × L^−1^;, the inset images are the dispersion of Fe_3_O_4_-CTA and Fe_3_O_4_15-*g*-P(BA65-*r*-TBEA20) in 1,4-dioxane and (**B**) FTIR spectrum of Fe_3_O_4_15-*g*-P(BA65-*r*-TBEA20).

The CTA-functionalized Fe_3_O_4_ NPs (i.e., Fe_3_O_4_-CTA) were exploited to mediate the RAFT copolymerization of *n*-butyl acrylate (BA) with 2-(3-tert-butyl-3-ethylureido)ethyl acrylate (TBEA) to obtain P(BA-*r*-TBEA)-grafted Fe_3_O_4_ NPs [denoted Fe_3_O_4_-*g*-P(BA-*r*-TBEA)]. By adjusting the mass ratios of Fe_3_O_4_-CTA to the monomers (i.e., BA + TBEA), Fe_3_O_4_-*g*-P(BA-*r*-TBEA)s were synthesized with (i) a fixed content of Fe_3_O_4_ NPs (viz. 15 wt%) and variable contents of TBEA from 10 to 40 wt% and (ii) a fixed content of TBEA (viz. 20 wt%) and variable contents of Fe_3_O_4_-CTA from 5 to 20 wt%. The compositions of the products are presented in Tables S1 and S2. In all the cases, the polymerizations were performed for sufficiently long time (e.g., 24 h or longer) to attain sufficiently high conversions of monomers. A successful grafting of P(BA-*r*-TBEA) chains onto the surfaces of Fe_3_O_4_ NPs was evidenced by the observation that Fe_3_O_4_-*g*-P(BA-*r*-TBEA)s were readily dispersed in an organic solvent (e.g., 1,4-dioxane), in contrast to the case of pristine Fe_3_O_4_ NPs (Figure 1A). The surface graft polymerization allowed the measurement of Fe_3_O_4_ NPs through dynamic light scattering (DLS) since Fe_3_O_4_-*g*-P(BA-*r*-TBEA)s can be finely dispersed in organic solvents (e.g., 1,4-dioxane) (Figure 1A). Taking Fe_3_O_4_15-*g*-P(BA65-*r*-TBEA20) for instance, the Fe_3_O_4_ NPs were measured to have the size of *D*_h_ = 122 nm. The Fe_3_O_4_-*g*-P(BA-*r*-TBEA)s were also used for FTIR spectroscopy. As shown in Figure 1B, the P(BA-*r*-TBEA) chains are characterized with two bands at 1730 and 1640 cm^−1^. The former is assignable to the stretching vibration of carbonyl groups whereas the latter to that of urea groups. The results of DLS and FTIR spectroscopy demonstrated that Fe_3_O_4_-*g*-P(BA-*r*-TBEA)s were successfully synthesized.

### 3.2. Crosslinking of Fe_3_O_4_-g-P(BA-r-TBEA) with HUBs

In the Fe_3_O_4_-*g*-P(BA-*r*-TBEA)s, each TBEA structural unit contains a single hindered urea bond (i.e., mono-HUB). To crosslink the Fe_3_O_4_-*g*-P(BA-*r*-TBEA)s, BBED (i.e., a hindered diamine) was incorporated to start the exchange of HUBs between TBEA and BBED, generating the inter-chain crosslinking of Fe_3_O_4_-*g*-P(BA-*r*-TBEA). In the reactions of exchange, *tert*-butylethylamine (i.e., a hindered monoamine) was yielded. Owing to the lower boiling point (~85 °C) [45], *tert*-butylethylamine was readily removed from the system via a simple distillation. Notably, the initially homogenous mixtures were gradually gelled with the consecutive removal of *tert*-butylethylamine, indicating that crosslinking took place (Figure S4). The crosslinking is attributable to the formation of di-HUBs among P(BA-*r*-TBEA) chains. To confirm the formation of crosslinking networks through the inter-chain HUBs, the networks were de-crosslinked by adding excess hindered monoamine (viz. *N*-*tert*-butylethylamine) in the mixture of the Fe_3_O_4_15-*g*-P(BA65-*r*-TBEA20) network with 1,4-dioxane (Figure S5). It was seen that the network was gradually “dissolved”, indicating that the formation of inter-chain HUBs was responsible for the crosslinking. The suspension was then subjected to the DLS measurement, and the “recovered” Fe_3_O_4_ NPs had a size of *D*_h_ = 142 nm. Notably, this value was slightly higher than that with Fe_3_O_4_15-*g*-P(BA65-*r*-TBEA20). The slightly increased *D*_h_ value is attributable to the additional chains from the remaining di-HUB linkages. The crosslinked Fe_3_O_4_15-*g*-P(BA65-*r*-TBEA20) was further subjected to TGA measurements (Figure S3). All the networks showed similar TGA profiles, indicating that the incorporation of Fe_3_O_4_ NPs did not alter the decomposition mechanism. Notably, the networks without Fe_3_O_4_ NPs were degraded completely at 800 °C. In contrast, the Fe_3_O_4_ NP-containing networks displayed degraded residues. The fact that the yields of the degradation residues were quite close to the feed ratios of Fe_3_O_4_ NPs suggests that the RAFT polymerizations were almost undergone completion.

**Figure 2:**
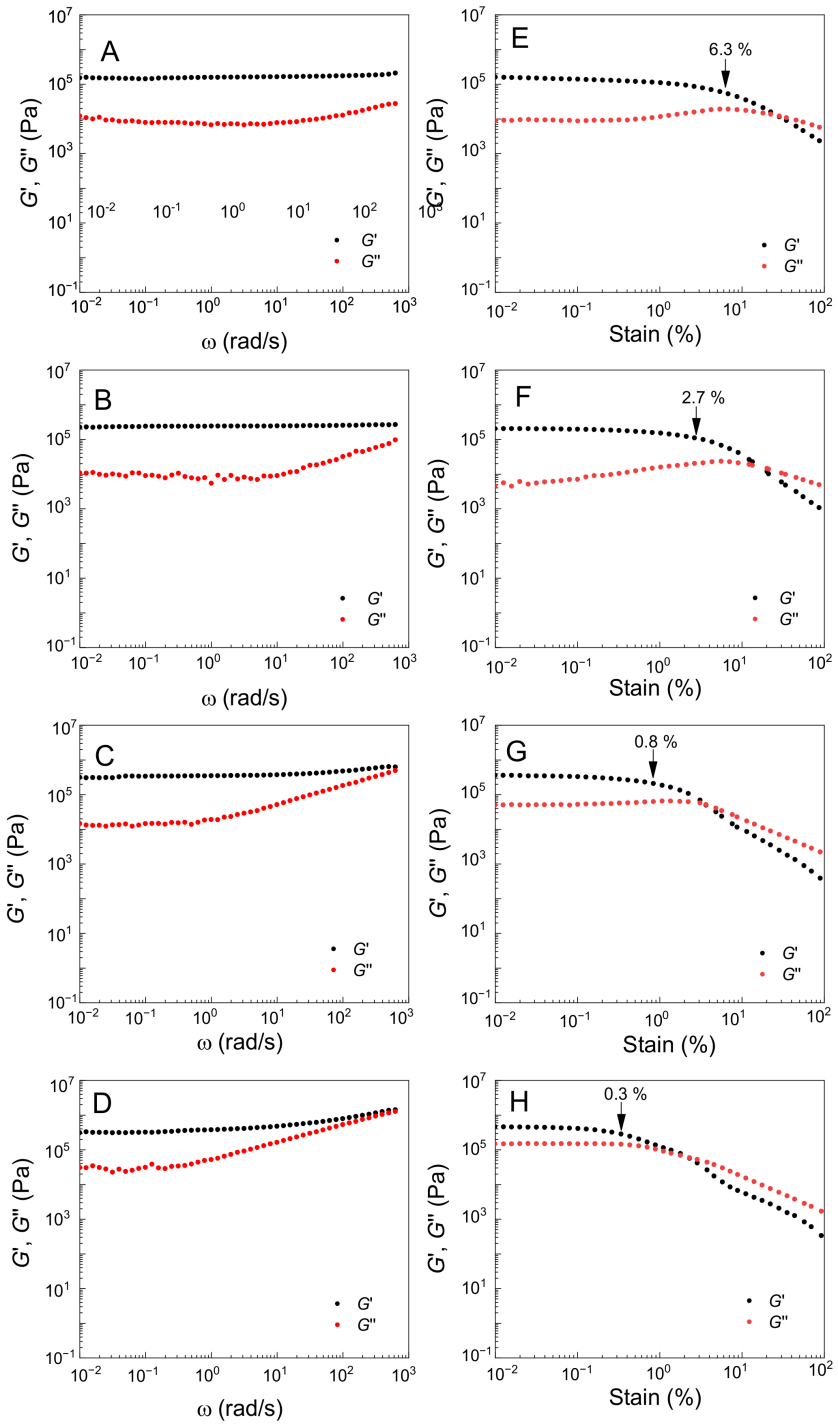
Angular frequency sweep (**left**) and strain sweep (**right**) curves of rheological measurements of the nanocomposites at 40 °C. (**A**,**E**) Fe_3_O_4_15*g*P(BA75*r*TBEA10), (**B**,**F**) Fe_3_O_4_15*g*P(BA65*r*TBEA20), (**C**,**G**) Fe_3_O_4_15*g*P(BA55*r*TBEA30) and (**D**,**H**) Fe_3_O_4_15*g*P(BA45*r*TBEA40).

The formation of Fe_3_O_4_-*g*-P(BA-*r*-TBEA) networks was readily confirmed by rheological analysis. In this work, the frequency and stain sweeps were carried out at 40 °C, which are shown in Figure 2. For the nanocomposites containing 15 wt% of Fe_3_O_4_ NPs and various contents of TBEAs, the frequency sweep curves showed that the storage moduli (*G*′) were significantly higher than the loss moduli (*G*″), indicating that the crosslinking was effectively formed in the nanocomposites. In the frequency range of 10^−2^ to 10^2^ rad × s^−1^, notably, the *G*′ values remained constant, irrespective of shear frequencies, suggesting that the crosslinked networks displayed the viscoelastic behavior of “perfect rubbers”. The *G*′ values increased as the TBEA content increased from 10 to 40 wt%, mainly attributable to the increase in the crosslinking densities. For the networks containing higher contents of TBEA (viz. 30 and 40 wt%), the *G*″ values rapidly increased with frequency. When the TBEA content was 40 wt%, the *G*′ and *G*″ curves intersected at 2 × 10^2^ rad × s^−1^, implying that the networks gained plasticity at the shear frequency. The plasticity resulted from the dynamic exchange of HUBs. From the strain sweep curves, all the samples displayed so-called strain-induced softening behavior, i.e., the *G*′ values dropped, whereas the *G*″ values increased at certain strain amplitudes. The decrease in *G*′ values reflects the destruction of the network structures under shear with sufficiently high strains. Notably, the strain amplitudes at which the *G*′ values declined significantly decreased from 6.3 to 0.3% with increasing the contents of TBEA from 10 to 40 wt%. The decreased strain amplitudes are mainly attributed to the increase in the contents of HUBs. The higher the content of HUBs, the more intense the exchange reaction.

**Figure 3:**
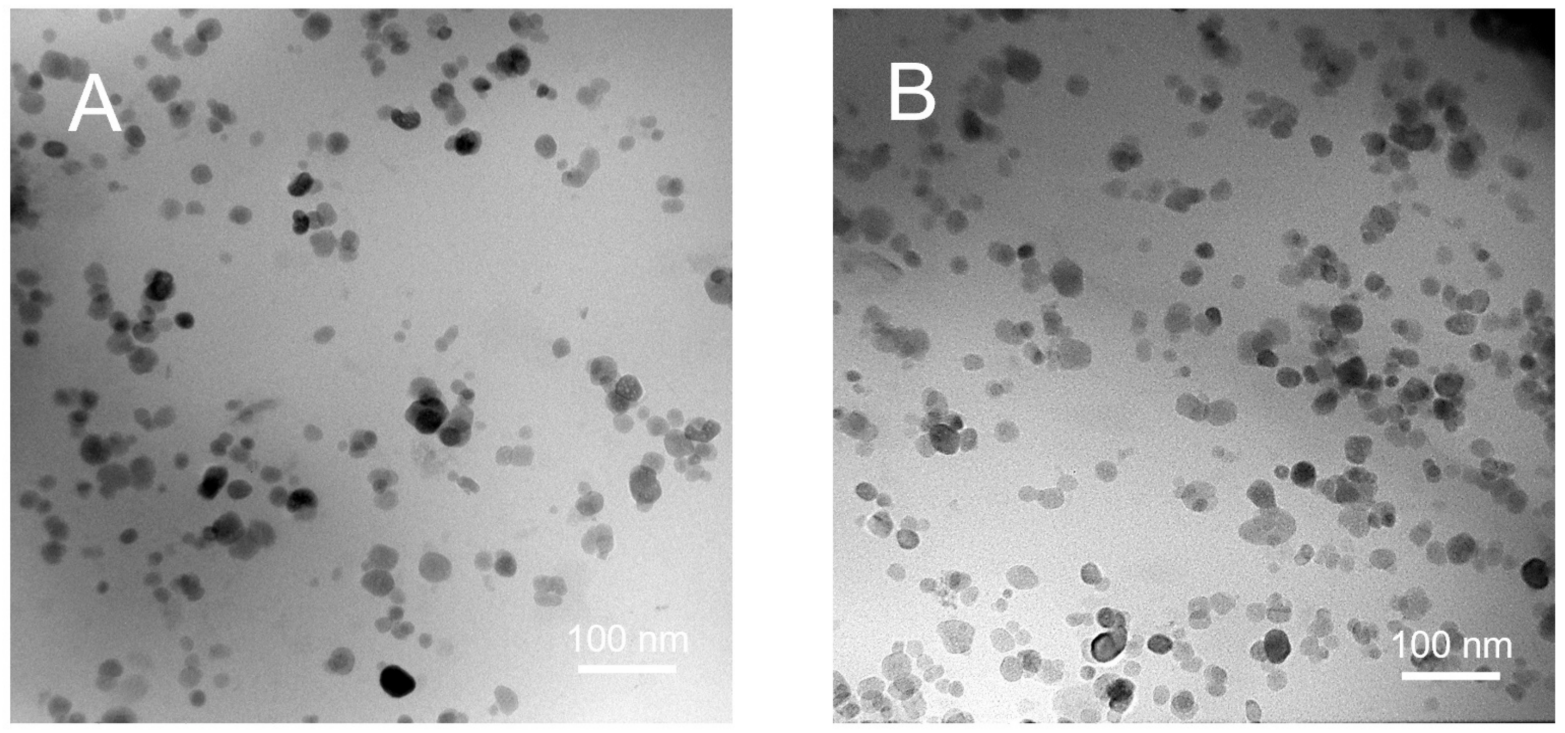
TEM images of (**A**) Fe_3_O_4_5-*g*-P(BA75-*r*-TBEA20) nanocomposite and (**B**) Fe_3_O_4_15-*g*-P(BA65-*r*-TBEA20) nanocomposite.

Transmission electron microscopy (TEM) was subsequently utilized to observe the morphologies of the networks. The samples were first frozen and grinded into powders in liquid nitrogen. The as-obtained powders were then dispersed in ethanol and dropped on the cupper grids. After evaporating the solvent, the morphologies of the samples were observed. Figure 3 shows the TEM images of the nanocomposites containing 5 and 15 wt% Fe_3_O_4_ NPs, respectively. The microphase-separated structure was observed in both cases. The Fe_3_O_4_ NPs, with the size in diameter from 20 to 40 nm, were uniformly dispersed in the P(BA-*r*-TBEA) matrix. For the networks containing higher contents of Fe_3_O_4_ NPs, slight aggregation of the Fe_3_O_4_ NPs was observed, which was a result of the strong magnetic dipolar interactions of Fe_3_O_4_ NPs. To further confirm the dispersion of Fe_3_O_4_ NPs in the polymer matrix, we examined the distribution of iron element in the samples through EDS measurements. As shown in the mapping image of iron element of Fe_3_O_4_15-*g*-P(BA65-*r*-TBEA20) nanocomposite (Figure S6), iron element was homogenously distributed in the matrix of P(BA-r-TBEA) and no obvious aggregation at the micrometer scale occurred. Both TEM and EDS results demonstrate that the nanocomposites were successfully obtained. The Fe_3_O_4_ NPs also acted as the physical crosslinking sites in the P(BA-*r*-TBEA) matrix. Therefore, the Fe_3_O_4_-*g*-P(BA-*r*-TBEA) nanocomposites feature both chemical crosslinking with HUBs and physical crosslinking from the inter-particle interactions of Fe_3_O_4_ NPs. This dual crosslinking significantly influenced the properties of the organic–inorganic nanocomposites.

### 3.3. Thermal and Mechanical Properties

**Figure 4:**
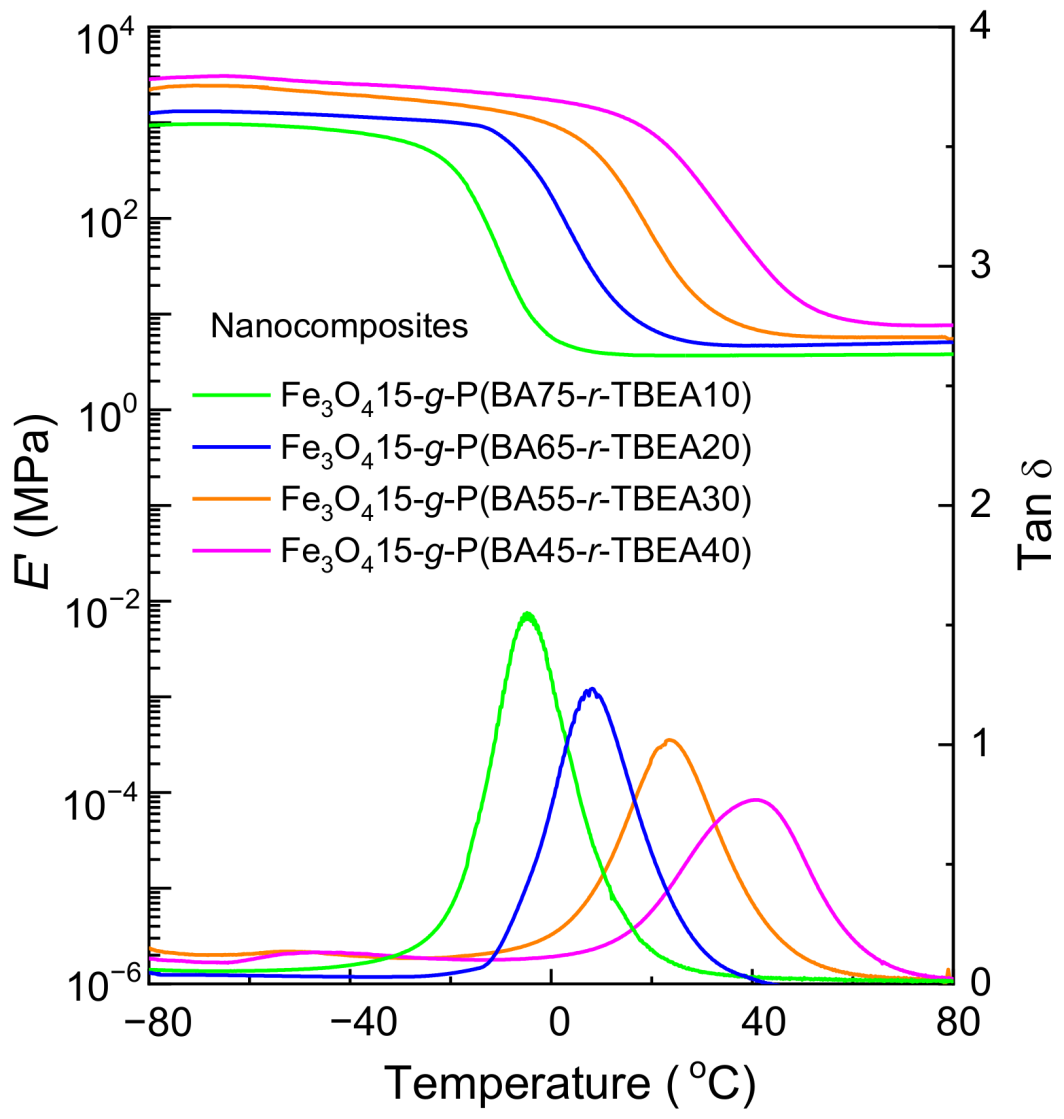
DMTA curves of the Fe_3_O_4_-*g*-P(BA-*r*-TBEA) nanocomposites.

The thermal properties of the Fe_3_O_4_-*g*-P(BA-*r*-TBEA) nanocomposites were investigated by dynamic mechanical thermal analysis (DMTA) (Figure 4). For the nanocomposites with a fixed Fe_3_O_4_ content and different TBEA content, the *T*_g_s exhibited a marked increase in correlation with the TBEA contents. For instance, with 10 wt% of TBEA, *T*_g_ was −4.6 °C, while with 40 wt% of TBEA, *T*_g_ was increased to 40.5 °C. The increase in *T*_g_ was ascribed to the enhancement of crosslinking densities. Higher contents of TBEA in the networks led to denser networks, which significantly restricted the chain segmental motion, resulting in higher *T*_g_’s. The increase in the crosslinking density was also indicated by the increase in *E*′ values with the TBEA contents.

**Figure 5:**
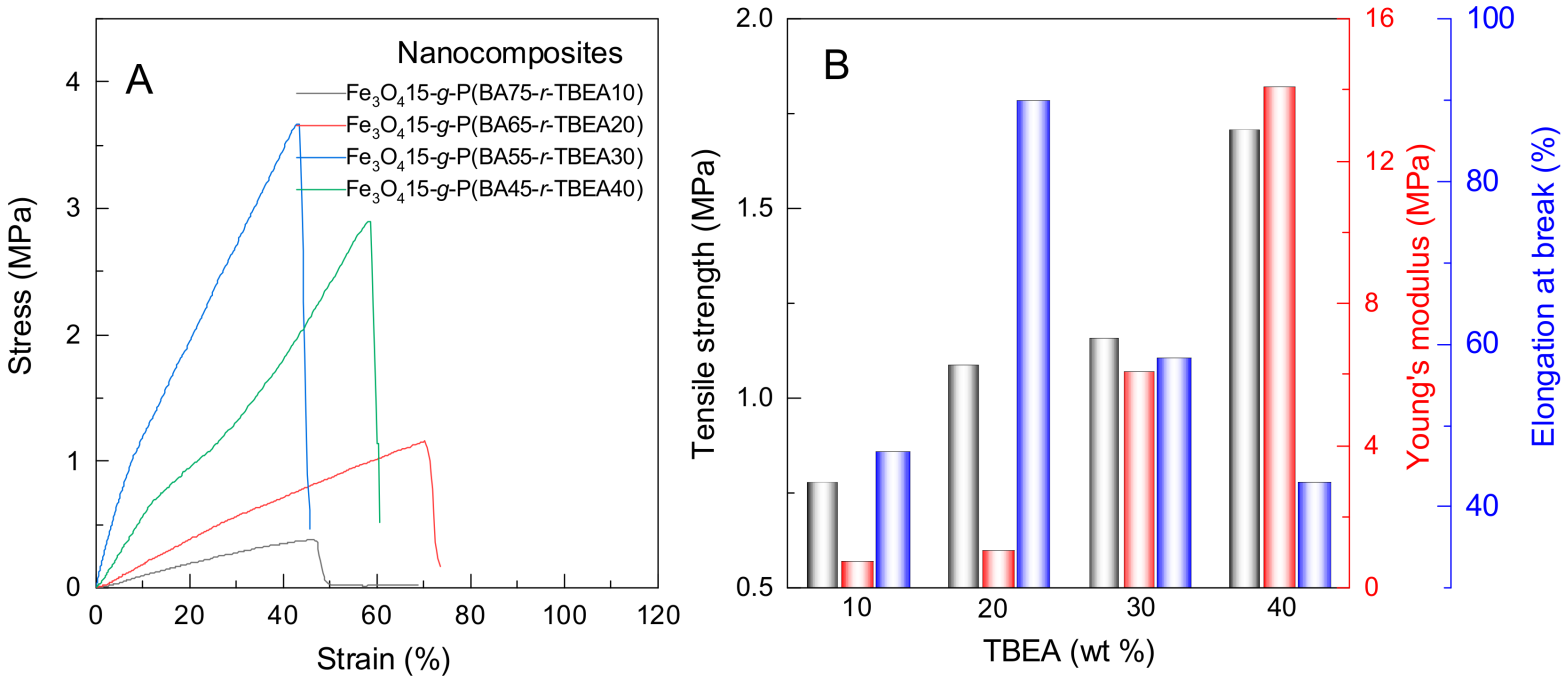
Stress–strain curves (**A**) and the mechanical parameters (**B**) of the Fe_3_O_4_-*g*-P(BA-*r*-TBEA) nanocomposites with different TBEA content.

The tensile tests on the Fe_3_O_4_-*g*-P(BA-*r*-TBEA) nanocomposites were conducted to study their mechanical properties (Figure 5). For the nanocomposites with 15 wt% of Fe_3_O_4_ NPs, both tensile strength and Young’s moduli showed an increasing tendency with the TBEA contents. The enhancement of crosslinking densities is favorable for improving the above mechanical parameters. It was noteworthy that there was a maximum value for the elongation at break (*ε*_b_ ≈ 72%) when the TBEA content was 20 wt%. It is proposed that there were two opposite factors that affected the elongations at break. On the one hand, the increased TBEA contents led to the increase in crosslinking densities and thus the elongation at break accordingly decreased. On the other hand, the increase in TBEA contents resulted in the increase in the contents of HUBs, causing the increase in self-adaptivity in response to external stretching. Therefore, when the samples were stretched, the continuous break and reform of networks increased the elongation at break. The combination of these two opposite effects resulted that when the TBEA content was 20 wt%, the nanocomposite displayed the maximum elongation at break. When the TBEA content was relatively high (viz. 30 and 40 wt%), the elongation at break displayed a declining tendency due to the increase in crosslinking densities.

### 3.4. Magnetic Properties

**Figure 6:**
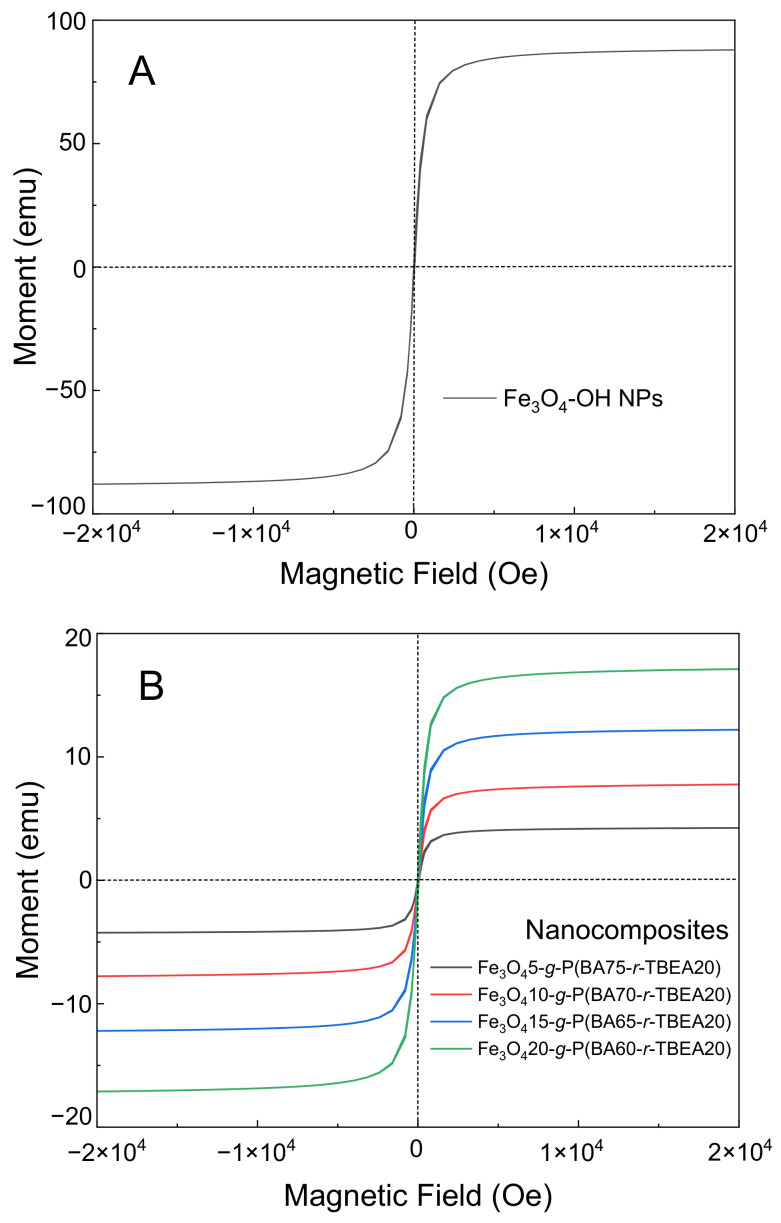
Magnetic curves of (**A**) Fe_3_O_4_-OH NPs and (**B**) Fe_3_O_4_-*g*-P(BA-*r*-TBEA) nanocomposites with different Fe_3_O_4_ content.

The magnetic properties of Fe_3_O_4_-*g*-P(BA-*r*-TBEA) nanocomposites were examined by the use of a vibrating-sample magnetometer (VSM). All the nanocomposites reached their saturation magnetization, representing the maximum magnetization intensity when the atom magnetic moments align in the same direction (Figure 6). The saturation magnetization of pristine Fe_3_O_4_ NPs was 88.18 emu × g^−1^, consistent with the literature values [15]. For the Fe_3_O_4_-*g*-P(BA-*r*-TBEA) nanocomposites containing 5, 10, 15 and 20 wt% Fe_3_O_4_ NPs, the saturation magnetization values were 4.42, 8.06, 12.18 and 17.39 emu × g^−1^, respectively, which were slightly lower than the theoretical values. It was proposed that two factors influenced the saturation magnetization. On the one hand, the surface graft copolymerization hindered the rotation of the magnetic moment along the direction of the magnetic field, reducing the saturation magnetization. On the other hand, the uniform dispersion of Fe_3_O_4_ NPs in the P(BA-*r*-TBEA) matrix facilitated their motion. The accelerated movement of Fe_3_O_4_ NPs promoted their alignment along the direction of the applied magnetic field, leading to an enhancement in saturation magnetization [16]. Ultimately, the first factor had a higher impact on the saturation magnetization. Additionally, the magnetization curves showed no obvious hysteresis loop and remanence, suggesting the superparamagnetic nature of Fe_3_O_4_-*g*-P(BA-*r*-TBEA) nanocomposites.

### 3.5. Reprocessing Properties

**Figure 7:**
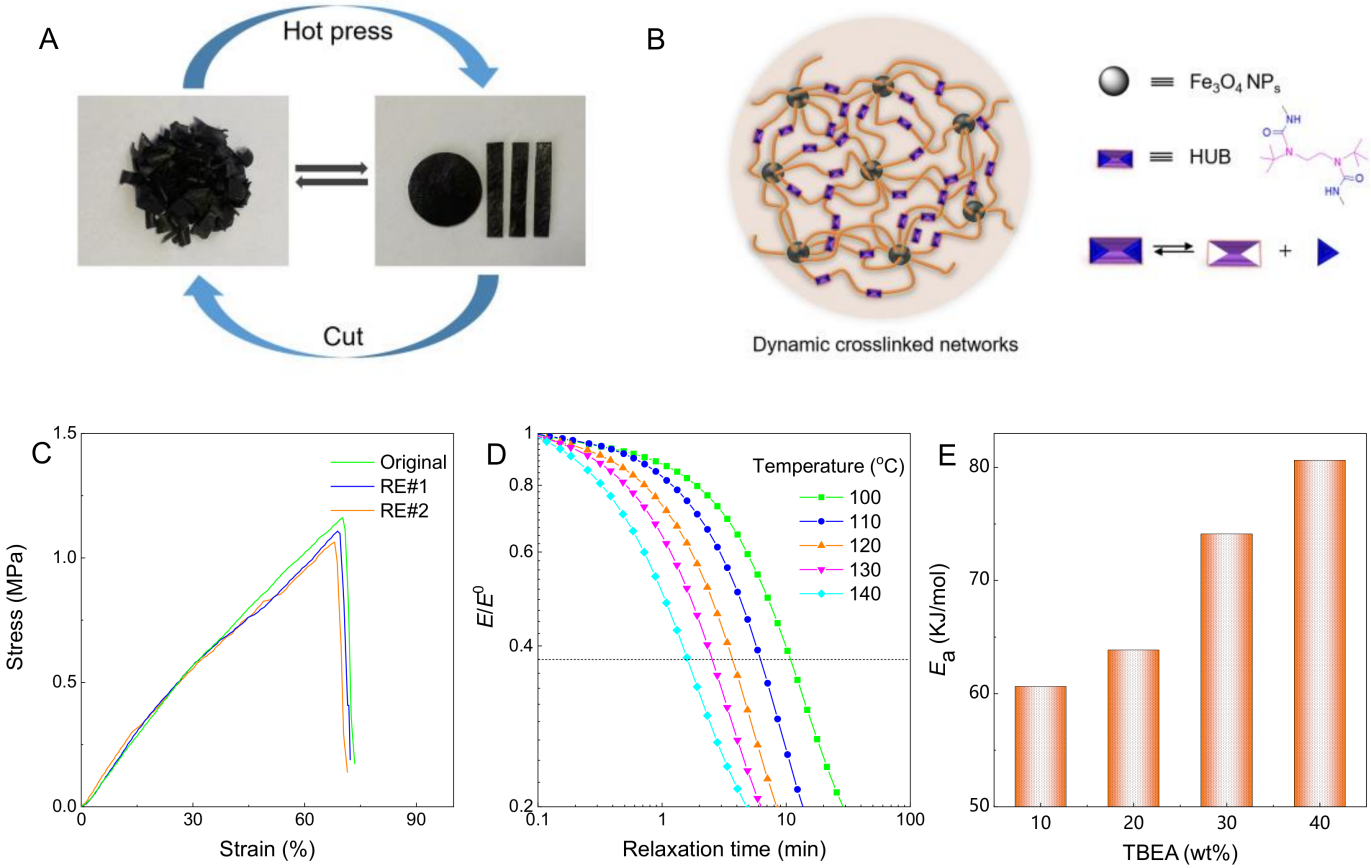
(**A**) The reprocessing of Fe_3_O_4_-*g*-P(BA-*r*-TBEA) nanocomposites, (**B**) the depiction of dynamic exchange, (**C**) stress-strain curves of the Fe_3_O_4_15-*g*-P(BA65-*r*-TBEA20) network before and after reprocessing, (**D**) stress relaxation curves of Fe_3_O_4_15-*g*-P(BA65-*r*-TBEA20) nanocomposite at variable temperature and (**E**) *E*_a_s of Fe_3_O_4_-*g*-P(BA-*r*-TBEA) nanocomposites with TBEA content.

The reprocessing experiments were implemented by cutting Fe_3_O_4_-*g*-P(BA-*r*-TBEA) nanocomposites into pieces and then compression molding at 100 °C for 30 min (Figure 7A). The compact block samples with variable shapes were obtained. The good reprocessing properties are ascribed to the exchange reaction of the HUBs (Figure 7B). When subjected to external physical (force) or temperature stimuli, the Fe_3_O_4_-*g*-P(BA-*r*-TBEA) nanocomposites displayed the self-adaptivity along with the external stretching, which resulted from the frequent break and reform of HUBs. To assess the extent of recovery of the mechanical properties after reprocessing, tensile tests were performed on the reprocessed nanocomposites. Representatively shown in Figure 7C are the strain–stress curves of Fe_3_O_4_15-*g*-P(BA65-*r*-TBEA20), and the stress-strain curves of the other specimens are shown in Figure S7. The original and reprocessed samples exhibited almost the the same strain–stress curves, that is, the mechanical parameters (e.g., tensile strength, Young’s modulus and elongation at break) did not change significantly. Here, the area under the stress-strain curves is defined as the fracture energy, allowing the calculation of the recovery degree (*F*) of mechanical properties in terms of the following equation [15]:

(1) $$F=\frac{\int_{0}^{r}\sigma (\varepsilon )d\varepsilon }{\int_{0}^{u}\sigma (\varepsilon )d\varepsilon }\times 100\%$$

where *σ* and *ε* represent stress and strain. The superscript “*u*” and “*r*” represent the original and reprocessed samples. All the *F* values of Fe_3_O_4_-*g*-P(BA-*r*-TBEA) nanocomposites exceeded 90%, revealing that the Fe_3_O_4_-*g*-P(BA-*r*-TBEA) networks possessed the excellent reprocessability.

To understand the effect of crosslinking densities on the exchange reaction of HUBs, the nanocomposites were subjected to stress relaxation tests at variable temperatures. Figure 7D shows the stress relaxation curves of Fe_3_O_4_15-*g*-P(BA65-*r*-TBEA20) and those of the other samples are shown in Figure S8. It is seen that the stress can be released quickly for all the samples, suggesting that the dynamic exchange of HUBs indeed took place in these networks. For the network without Fe_3_O_4_ [viz. P(BA-*r*-TBEA)], notably, the stress was released completely (Figure S9). In contrast, the stress cannot be decayed to zero for the Fe_3_O_4_-*g*-P(BA-*r*-TBEA) nanocomposites. It was proposed that the Fe_3_O_4_ NPs can behave as the crosslinking sites to generate some permeant crosslinking. Assuming that the dynamic chemistry responsible for the reprocessing properties of the nanocomposites follows an Arrhenius model [15], the following equation applies:

(2) $$\tau =\tau_{0}\exp(-\frac{E_{a}}{RT})$$

where *τ* denotes the relation time, at which the stress is relaxed to 1/*e* of the initial value, and *A*, *T* and *R* represent the pre-exponential factor, absolute temperature and universal gas constant, respectively. The activation energy can be calculated by linear plots of the ln(*τ*) versus 1000/*T* curves (Figure 7E). For the Fe_3_O_4_-*g*-P(BA-*r*-TBEA) nanocomposites containing 15 wt% of Fe_3_O_4_ NPs, the *E*_a_ values showed an increased tendency from 60.61 to 80.62 kJ mol^−1^ with increasing the TBEA contents from 10 to 40 wt%. It is proposed that the stress relaxation behavior was influenced by two opposite factors. The increase in HUB contents promoted the stress relaxation. Conversely, the HUBs served as the crosslinking sites that restricted the stress relaxation. With increasing the HUB contents, the crosslinking densities increased. Herein, the effect of the crosslinking density dominated in the *E*_a_ values. Overall, the unique structural and dynamic characteristics would endow Fe_3_O_4_-*g*-P(BA-*r*-TBEA) networks with mechanically adaptive properties.

### 3.6. Multi-Stimuli-Responsive Shape Memory Properties

The presence of crosslinking structures enabled the Fe_3_O_4_-*g*-P(BA-*r*-TBEA) nanocomposites to exhibit shape memory properties with the *T*_g_s serving as the shape transition temperatures (*T*_tr_s). The Fe_3_O_4_ NPs can absorb near-infrared (NIR) light and transform it into thermal energy [55,56]. In addition, the Fe_3_O_4_ NPs served as internal mini-antennas to transform the electromagnetic energy to inductive Joule heat in an alternating field [57,58,59]. The unique properties of Fe_3_O_4_ NPs enable the networks to not only exhibit the common thermally induced shape memory performance incontact mode (viz. hot stage or oven) but also to undergo shape memory properties in noncontact modes (e.g., near-infrared light, magnetic field).

**Figure 8:**
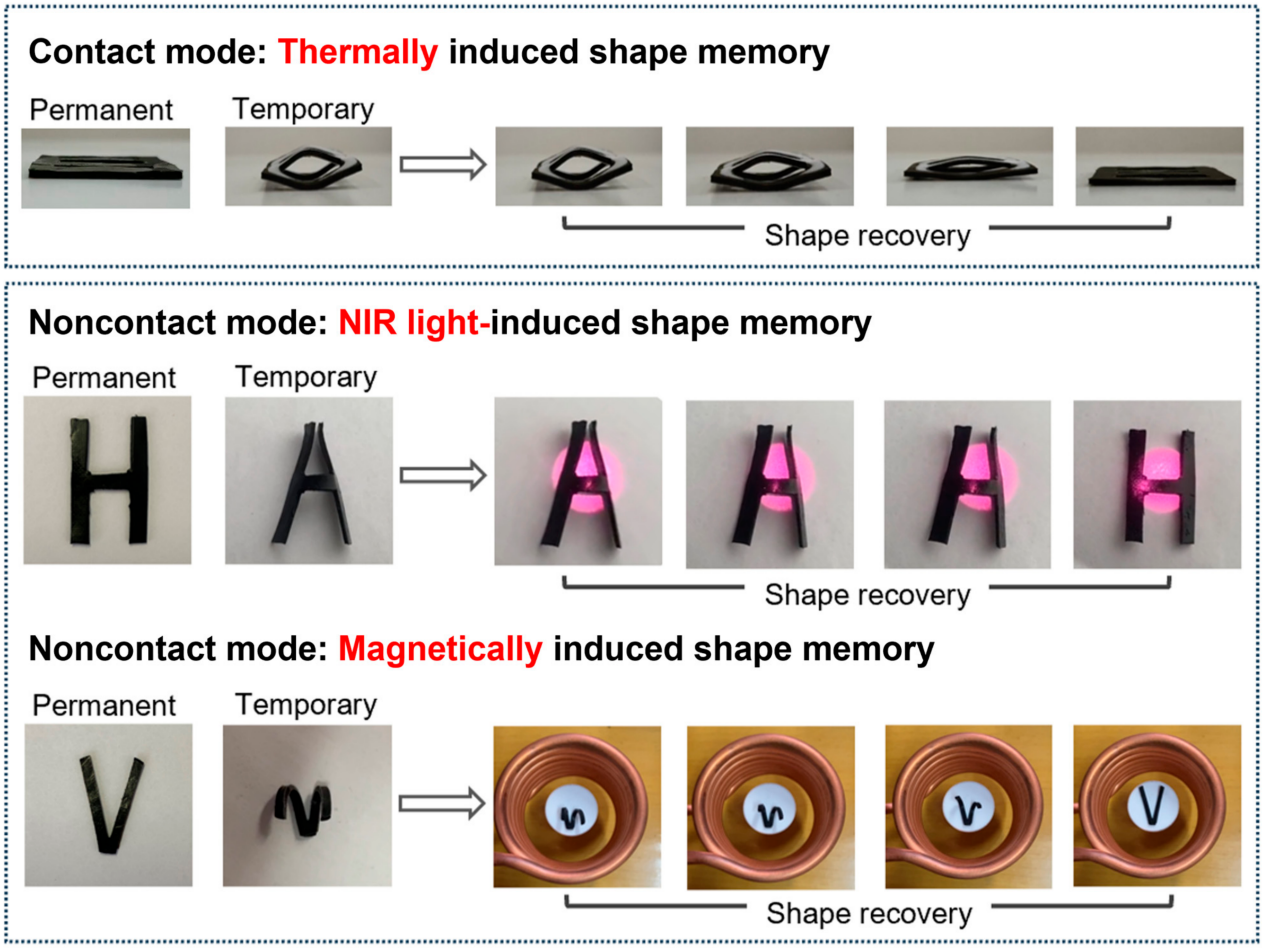
Thermally, NIR light- and magnetically induced shape memory process of Fe_3_O_4_15-*g*-P(BA65*-r*-TBEA20) nanocomposite.

The contact mode shape memory behaviors were investigated, as shown in Figure 8. The samples with a permanent shape (“closed eye”) were first deformed into a temporary shape (“opened eye”) on a heat stage at 40 °C for 5 min, and this shape was fixed by transferring the specimen to the freezer at −40 °C for 10 min. Subsequently, the samples were then transferred to the hot stage at 40 °C. Within a short time, the temporary shape (“opened eye”) recovered to the original shape (“closed eye”). By comparison, notably, the shape recovery was accelerated as the Fe_3_O_4_ NP content increased. The accelerated shape recovery rate can be attributed primarily to the increase in the crosslinking densities.

The noncontact mode shape memory behaviors were studied under near-infrared (NIR) light and magnetic fields. The Fe_3_O_4_-*g*-P(BA-*r*-TBEA) nanocomposites were subjected to the irradiation with an NIR light at the wavelength of λ = 808 nm, while the surface temperature was concurrently monitored using an infrared camera (Figure S10). The nanocomposite without Fe_3_O_4_ NPs exhibited only a slight temperature increase (viz. 5 °C) after irradiating for 60 s. However, the surface temperature of all the Fe_3_O_4_-*g*-P(BA-*r*-TBEA) nanocomposites significantly increased. When the Fe_3_O_4_ content was 20 wt%, the surface temperature reached 98 °C. The photothermal response of the nanocomposites was employed to activate the shape memory characteristics under near-infrared irradiation. Taking Fe_3_O_4_15-*g*-P(BA65-*r*-TBEA20) as an example, the sample was first cut into an “H” shape and deformed into an “A” shape at 40 °C. The “A” shape was then fixed by quickly cooling to −40 °C. The specimen was irradiated with the NIR light. Upon irradiating, the specimen recovered to its original “H” shape completely (Figure 8).

The nanocomposites were irradiated under magnetic field and the surface temperature was also monitored with an infrared camera (Figure S11). For the nanocomposite without Fe_3_O_4_ NPs, the temperature was almost unchanged. However, the surface temperature of Fe_3_O_4_-*g*-P(BA-*r*-TBEA) nanocomposites significantly increased. For the nanocomposite with the Fe_3_O_4_ NP content of 20 wt%, the surface temperature rose to 54 °C after irradiating for 120 s. The magnetic–thermal behavior was also employed to trigger the shape memory properties. Representatively shown in Figure 8 are the photographs of the Fe_3_O_4_15-*g*-P(BA65-*r*-TBEA20) nanocomposite for the magnetic field-triggered shape memory test. First, the sample was cut into a “V” shape and deformed into an “M” shape at 40 °C, which was fixed by cooling to −40 °C. Afterwards, the V-shaped specimen was quickly transformed in a copper-induced magnetic coil. The thermal energy was generated due to the Brownian and Néel relaxation losses of the Fe_3_O_4_ nanoparticles in the alternating magnetic field, which caused the temperature to exceed the *T*_g_ value of the nanocomposites [60]. The sample completely recovered to its original “V” configuration.

**Figure 9:**
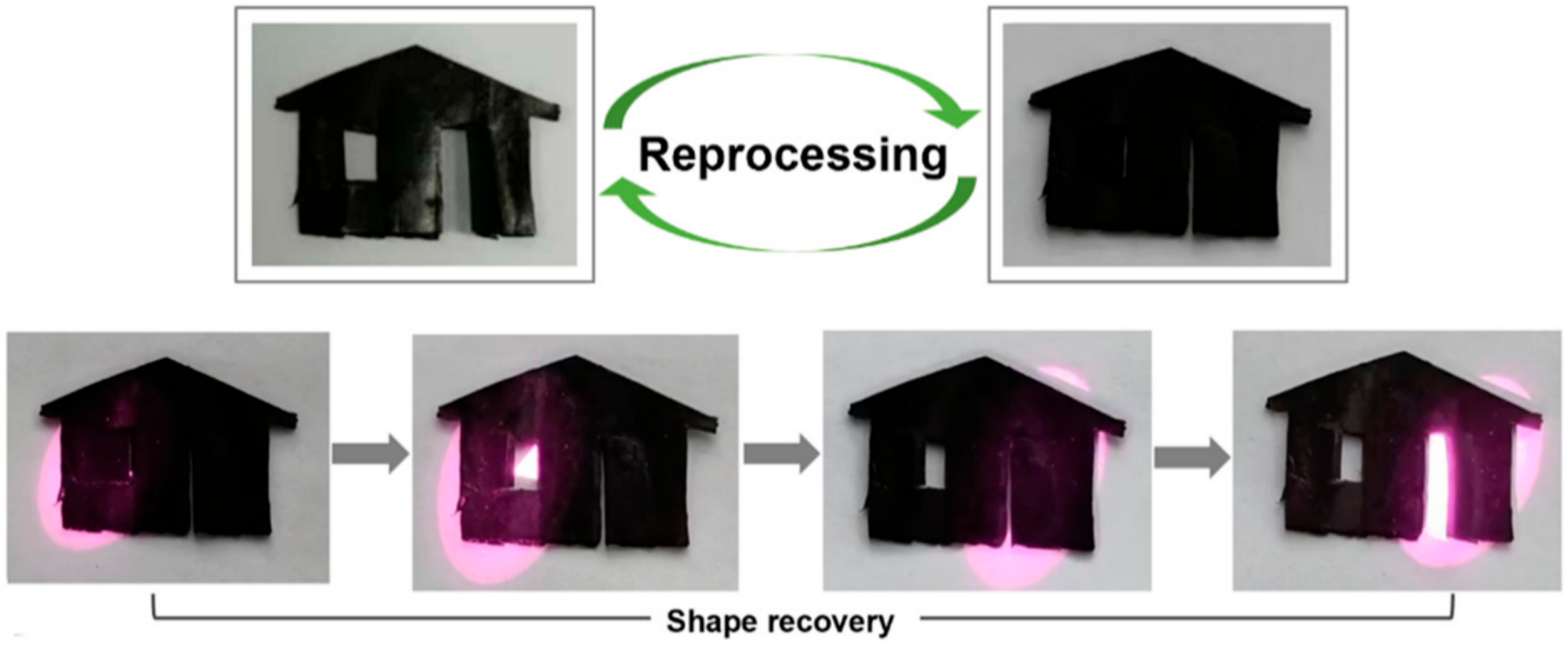
Photographs for shape reprocessing and shape memory test of Fe_3_O_4_15-*g*-P(BA65*-r*-TBEA20) nanocomposite.

The formation of dynamically crosslinked networks provided the possibility for the shape reprogramming of Fe_3_O_4_-*g*-P(BA-*r*-TBEA) networks. Taking the Fe_3_O_4_15-*g*-P(BA65-*r*-TBEA20) network for example, a “house” with a “closed window” and “closed door” was made by cutting the sample at room temperature (Figure 9). The window and door were opened under an external force at 100 °C, and this state was fixed by annealing the specimen for 1h. Notably, a new permanent shape was generated. This involved a rearrangement of the network through the dynamic exchange of HUBs. In addition, the internal stress was relaxed through the reversible rearrangement of the dynamic covalent bonds, leading to the absence of elastic entropy that would otherwise facilitate the reversion of the specimen to its permanent shape. As a result, a new permanent shape was established. The specimen with the newly reprogrammed configuration could also be deformed into a temporary shape (viz. closed “window” and “door”), which was then fixed by cooling it to −40 °C for 10 min. When the “window” was irradiated by the laser, notably, the door was automatically opened, that is, the original shape was recovered. Similarly, the “door” could also be opened with irradiating by the laser. Regarding the rapid transformation and shape locking behavior, the Fe_3_O_4_-*g*-P(BA-*r*-TBEA) nanocomposites exhibited significant potential as advanced shape memory materials.

## 4. Conclusions

In summary, Fe_3_O_4_-grafted copolymers [Fe_3_O_4_-*g*-P(BA-*r*-TBEA)s] were synthesized via the surface-initiated RAFT polymerization approach. The Fe_3_O_4_-*g*-P(BA-*r*-TBEA)s were readily crosslinked through the dynamic exchange reaction with BBED. By changing the contents of Fe_3_O_4_ NPs and TBEA, a series of dynamically crosslinked networks with HUBs were successfully constructed and Fe_3_O_4_ NPs displayed fine dispersion in the continuous P(BA-*r*-TBEA) matrix. It was found that the thermal and mechanical properties can be customized by varying the contents of Fe_3_O_4_ NPs and/or TBEA. The introduction of HUBs endowed the networks with excellent reprocessability. The photo- and magnetic–thermal effect of Fe_3_O_4_ NPs endowed the networks with shape memory performance driven by near-infrared light and magnetic fields.

## Data Availability

Data are contained within the article and Supplementary Materials.

## Supplementary Materials

The following supporting information can be downloaded at: https://www.mdpi.com/article/10.3390/polym16182638/s1, Scheme S1: Synthesis of 2-(3-*tert*-butyl-3-ethylureido)ethyl acrylate (TBEA); Table S1: Compositions of Fe_3_O_4_-*g*-P(BA-*r*-TBEA)s with different TBEA contents; Table S2: Compositions of Fe_3_O_4_-g-P(BA-*r*-TBEA)s with different Fe_3_O_4_ NPs; Figure S1: ^1^H NMR spectrum of TEBA; Figure S2: FTIR spectra of Fe_3_O_4_-OH, Fe_3_O_4_-NH_2_ and Fe_3_O_4_-CTA; Figure S3: TGA curves of Fe_3_O_4_ NPs and nanocomposites; Figure S4: Photographs of the mixture of 1,2-bis(tert-butyl)ethylenediamine) with 10 wt% of Fe_3_O_4_15-*g*-P(BA65-*r*-TBEA20) copolymer before and after crosslinking; Figure S5: Photographs of solubility tests for Fe_3_O_4_15-*g*-P(BA65-*r*-TBEA20) nanocomposite: (left) swollen with 1,4-dioxane; (right) held at room temperature for 24 h after N-tert-butylethylamine was added; Figure S6: Iron element mapping image of Fe_3_O_4_15-*g*-P(BA65-*r*-TBEA20) nanocomposite; Figure S7: Stress-strain curves of the Fe_3_O_4_-*g*-P(BA-*r*-TBEA) nanocomposites before and after reprocessing; Figure S8: Stress relaxation curves of nanocomposites: (A) Fe_3_O_4_15-*g*-P(BA75-*r*-TBEA10), (B) Fe_3_O_4_15-*g*-P(BA55-*r*-TBEA30) and (C) Fe_3_O_4_15-*g*-P(BA45-*r*-TBEA40); Figure S9: Stress relaxation curves of P(BA80-*r*-TBEA20) nanocomposite; Figure S10: Surface temperature images of nanocomposites: (A) P(BA80-*r*-TBEA20), (B) Fe_3_O_4_5-*g*-P(BA75-*r*-TBEA20), (C) Fe_3_O_4_10-*g*-P(BA70-*r*-TBEA20), (D) Fe_3_O_4_15-*g*-P(BA65-*r*-TBEA20), (E) Fe_3_O_4_20-*g*-P(BA60-*r*-TBEA20), (F) surface temperature profiles of the nanocomposites as function of the time; Figure S11: Surface temperature images of nanocomposites under the magnetic field: (A) Fe_3_O_4_20-*g*-P(BA60-*r*-TBEA20), (B) Fe_3_O_4_15-*g*-P(BA65-*r*-TBEA20), (C) Fe_3_O_4_10-*g*-P(BA70-*r*-TBEA20), (D) Fe_3_O_4_5-*g*-P(BA75-*r*-TBEA20), (E) surface temperature profiles of the nanocomposites as function of the time.
