# Supplementary material for: Nanocomposites of Poly(n-Butyl Acrylate) with Fe3O4: Crosslinking with Hindered Urea Bonds, Reprocessing and Related Functional Properties
Source: Polymers (Basel). 2024 Sep 18;16(18):2638. doi: 10.3390/polym16182638 (PMC11436229; doi:10.3390/polym16182638)
Supplement: Supplementary file 1 [file polymers-16-02638-s001.zip › polymers-3206025-supplementary.pdf]

SUPPORTING INFORMATION for

Nanocomposites of Poly(*n*-butyl acrylate) with Fe_3O_4: Crosslinking with Hindered Urea Bonds, Reprocessing and Related Functional Properties

Lei Li, Huaming Wang, Xibin Shen, Guohua Hang, Yuan Gao, Jiawei Hu, Sixun Zheng*

Department of Polymer Science and Engineering and the State Key Laboratory of Metal Matrix Composites, Shanghai Jiao Tong University, Shanghai 200240, P. R. China

* Corresponding author, *email:* szheng@sjtu.edu.cn (S. Zheng)

TABLE OF CONTENTS

1. ***Materials***……………………………………………………………………….S3

2. ***Synthesis of pristine Fe_3O_4, aminopropyl- and trithiocarbonate-functionalized Fe_3O_4 NPs***………………………………………………………………….S3

3. ***Synthesis of 2-(3-tert-butyl-3-ethylureido)ethylacrylate***……………..………S4

4. **Measurements and Techniques**…………………….………………………S4

4.1. *Nuclear Magnetic Resonance (NMR) Spectroscopy*……………………..…….S4

4.2. *Fourier Transform Infrared (FTIR) Spectroscopy*…………………………S5

4.3. *Thermal Gravimetric Analysis (TGA)*……………………….......................S5

4.4. *Transmission Electron Microscopy (TEM)* ……………………..................S5

4.5. *Scanning Electron Microscopy (SEM)* …………………….........................S5

4.6. *Rheological Measurements*……………………………..…………………S6

4.7. *Dynamic Mechanical Thermal Analysis (DMTA)*…………………….……S6

4.8. *Magnetic Analysis*…………………………...S6

4.9. *Stress Relaxation Test*…………..…………………………..........................S6

4.10. *Tensile Mechanical Tests*…. …………………….......................................S6

5. **Schemes, Tables and Figures**……………………......................................S7

1. *Materials*

3-Aminopropyltriethoxysilane (APTES) was purchased from TCI Co. Shanghai, China and used as received. Ferric chloride ($FeCl_3 \cdot 6H_2O$), ferrous sulfate ($FeSO_4 \cdot 7H_2O$) and ammonium hydroxide were purchased from Sinopharm Chemical Reagent Co., Shanghai, China. Lithium bromide, 4-dimethylaminopyridine (DMAP) and 1-(3-dimethylaminopropyl)-3-ethylcarbodiimide hydrochloride (EDC) were purchased from Acros Co., China. All the organic solvents were purchased from Titan Co., China.

2. *Synthesis of pristine Fe_3O_4, aminopropyl- and trithiocarbonate-functionalized Fe_3O_4 NPs*

Plain Fe_3O_4 NPs (Fe_3O_4-OH NPs) were synthesized with a chemical co-precipitation technique. Typically, to a flask $FeCl_3 \cdot 6H_2O$ (10.820 g), $FeSO_4 \cdot 7H_2O$ (5.560 g) and the distilled water (200 mL) were charged. This system was purged with argon for 30 min and then 200 mL of aqueous ammonia solution (0.7 mol/L) was dropwise added within 60 min. The reaction was performed at 60 °C for additional 60 min. The precipitates were collected and washed with deionized water three times. After dried *in vacuo* at 30 °C for 24 hours, the product (8.290 g) was obtained with the yield of 95.1%. FTIR (cm^{-1}, KBr window): 3200 (O-H), 1338 and 1632 (O-H from the $Fe_3O_4 \cdot H_2O$), 565 (Fe-O).

To a flask equipped with a magnetic stirrer, the above Fe_3O_4-OH NPs (1.000 g) and 300 mL of the mixture of ethanol with water (50:50 vol) were charged with vigorous stirring. This flask was subjected to an ultrasonic irradiation for 30 min to activate the surface of the Fe_3O_4 NPs. Thereafter, 3-aminopropyltriethoxysilane (APTES) (15.000 g) was added with vigorous stirring. The reaction was then performed at 50 °C for 7 hours. The surface-functionalized Fe_3O_4 NPs were collected and washed with deionized water three times. After dried *in vacuo* at 30 °C for 24 hours, the APTES-modified Fe_3O_4 NPs were obtained with the yield of 92%. FTIR (cm^{-1}, KBr window): 565 (Fe-O), 1338 and 1632 (O-H from the $Fe_3O_4 \cdot H_2O$), 3124 (N-H), 3200 (O-H).

The above Fe_3O_4-NH_2 NPs were used to react with DDMAT to obtain the trithiocarbonate-functionalized Fe_3O_4 NPs (Fe_3O_4-CTA NPs). To a flask, Fe_3O_4-NH_2 (3.000 g) and dichloromethane (50 mL) were charged; the mixture was treated

with ultrasonic irradiation for 30 min. Thereafter, DDMAT (1.120 g, 4.0 mmol), EDC (1.540 g, 8.0 mmol) and DMAP (0.300 g, 2.4 mmol) were added. At room temperature, the reaction was carried out for 36 h. After centrifugation, the product was washed with dichloromethane thrice until the supernatant liquid became colorless. After drying, the trithiocarbonate-functionalized Fe3O4 NPs were obtained with the yield of 95%. FTIR (KBr window, cm^{-1}): 3426 (O-H), 2926 (C-H of CH2), 2845 (C-H of CH_3), 1629 (>C=O), 1578 (C-N), 1116 (Fe-O-Si), 1013 (Si-O-Si) and 591 (Fe-O).

3. *Synthesis of 2-(3-tert-butyl-3-ethylureido)ethyl acrylate*

To a flask, 2-isocyanatoethyl acrylate (14.100 g, 10.0 mmol) was dissolved in 30 mL dichloromethane and the dichloromethane solution (20 mL) of *N-tert-butylethylamine* (8.716 g, 10.0 mmol) was added dropwise at 0 ^oC. After dropping, the reaction was performed for additional one hour. The crude compound was passed through a basic alumina column with ethyl acetate as the eluent. After rotary evaporation, the product [*viz.* 2-(3-tert-butyl-3-ethylureido)ethyl acrylate, TBEA] (21.360 g) was obtained with a 95% yield. ^1H NMR (ppm, $CDCl_3$): 6.68 (1H, -OCOCH = C$\boldsymbol{H}_2$), 6.06 (1H, -OCOC$\boldsymbol{H}$=CH_2), 5.75 (1H, -OCOCH=C$\boldsymbol{H}_2$), 4.77 (1H, -$OCH_2CH_2N\boldsymbol{H}$-), 4.16 (2H, -OC$\boldsymbol{H}_2CH_2$NH-), 3.52 (2H, -$OCH_2C\boldsymbol{H}_2$NH–), 3.28 (2H, -NHC$\boldsymbol{H}_2(CH_3)_3CH_3$), 1.28 [9H, -NC(C$\boldsymbol{H}_3)_3CH_3$], 1.26 [3H, -$NC(CH_3)_3C\boldsymbol{H}_3$]

4. *Measurements and Techniques*

4.1 Nuclear Magnetic Resonance (NMR) Spectroscopy

^1H NMR spectra were recorded with a Bruker Advance 500 spectrometer. Deuterium chloroform was used as the solvent.

4.2 Fourier Transform Infrared (FTIR) Spectroscopy

The FTIR measurements were conducted on a Perkin-Elmer Paragon 1000 Fourier transform spectrometer at room temperature (25 °C). The films of specimens were obtained *via* casting the chloroform solution of the samples (2 wt%) onto KBr windows. For the preparation of thermoset specimens, the

thermosets were granulated and the powder was mixed with KBr pellets to press into the small flakes for measurements. All the specimens were sufficiently thin to be within a range where the Beer-Lambert law is obeyed. In all cases 64 scans at a resolution of 2 cm^{-1} were used to record the spectra.

4.3 Thermal Gravimetric Analysis (TGA)

The TGA measurements were carried out on a TA Instrument Q5000 thermal gravimetric analyzer. The measurements were performed in nitrogen atmosphere from 40 °C to 800°C with a heating rate of 20 °C/min. Temperature of initial degradation (T_d) was taken as the onset temperature, at which 5 wt % of weight loss occurs. The content of CTA can be calculated according to the following equation:

$$A = \frac{M_1 - M_2}{W_{org}} \tag{S1}$$

where M_1 and M_2 are the residues for 1g of Fe_3O_4-OH NPs and Fe_3O_4-NH_2 NPs, and W_{org} was the mass weight of the organic components in the Fe_3O_4-NH_2 NPs.

4.4 Transmission Electron Microscopy (TEM)

Transmission electron microscopy (TEM) was performed on a JEOL JEM-2010 high-resolution transmission electron microscope at an acceleration voltage of 120 kV. The samples were first frozen and grinded into powders in liquid nitrogen. The as-obtained powders were then dispersed in ethanol and dropped on the cupper grides. After evaporating the solvent, the morphologies of the samples were observed.

4.5 Scanning Electron Microscopy (SEM)

The thermosets were fractured under cryogenic condition using liquid nitrogen. The fracture surfaces were coated with thin layers of gold. The specimens were examined with an Apreo 2S scanning electron micro-scope (SEM) at an activation

voltage of 5 kV. Qualitative and semiquantitative chemical analysis in the model of energy-dispersive X-ray spectroscopy (EDX) was performed with a ThermoNORAN model (Quest model) spectrometer.

4.6 Rheological Measurements

The rheological measurements were performed on a DHR-2 stress-controlled rheometer (TA, USA) at 40 °C. The linear viscoelastic (LVE) regime of each circular specimen was determined by applying strain sweeps at constant frequency of 1Hz.

4.7 Dynamic Mechanical Thermal Analysis (DMTA)

Dynamic mechanical properties were measured by a DMTA Q800 instrument to obtain the storage modulus and loss tangent (tan δ) messages. The measurements were performed in a stretch mode at a frequency of 1 Hz and a heating rate of 3 °C/min.

4.8 Magnetic Analysis

The magnetic properties were measured on a superconducting quantum interferometer device (SQUID) with vibrant measuring sample technology (VSM) (VSM, Lake Shore 7400) at room temperature. The range of the magnetic field is from -20 to 20k Oe.

4.9 Stress Relaxation Tests

Stress relaxation tests were performed with a TA Q800 dynamic mechanical thermal analyzer. The measurements were performed with a constant strain of 10 % at different temperature.

4.10 Tensile Mechanical Tests

Tensile mechanical tests were performed with a WDW-2 electron universal testing machine (Songdun Instruments Co. Ltd., Shanghai, China). The uniaxial stretching experiments were carried out at a loading rate of 50 mm/min at room temperature. For each sample, five parallel specimens were tested and then the average results were reported.

SCHEMES AND FIGURES

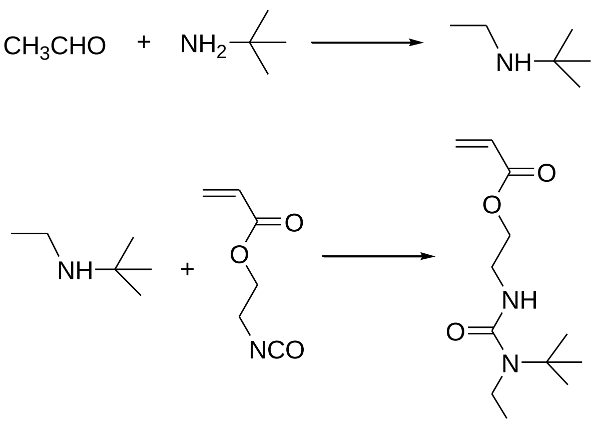

Scheme S1 Synthesis of 2-(3-*tert*-butyl-3-ethylureido)ethyl acrylate (TBEA)

Table S1. Compositions of Fe_3O_4-*g*-P(BA-*r*-TBEA)s with different TBEA contents

**Samples**	**Fe_3O_4 (wt%)^a**	**BA+TBEA (wt%)^a**	**TBEA (wt%)^a**	**Fe_3O_4 (wt%)^b**
$Fe_3O_4$15-*g*-P(BA75-*r*-TBEA10)	15	85	10	14.7
$Fe_3O_4$15-*g*-P(BA65-*r*-TBEA20)	15	85	20	15.3
$Fe_3O_4$15-*g*-P(BA55-*r*-TBEA30)	15	85	30	14.8
$Fe_3O_4$15-*g*-P(BA45-*r*-TBEA40)	15	85	40	15.1

a: from the feed ration, *b*: calculated according to TGA measurements

Table S2. Compositions of Fe_3O_4-*g*-P(BA-*r*-TBEA)s with different Fe_3O_4 NPs

**Samples**	**Fe_3O_4 (wt%)^a**	**BA+TBEA (wt%)^a**	**TBEA (wt%)^a**	**Fe_3O_4 (wt%)^b**
$Fe_3O_4$5-*g*-P(BA75-*r*-TBEA20)	5	95	20	4.6
$Fe_3O_4$10-*g*-P(BA70-*r*-TBEA20)	10	90	20	9.2
$Fe_3O_4$15-*g*-P(BA65-*r*-TBEA20)	15	85	20	15.3
$Fe_3O_4$20-*g*-P(BA60-*r*-TBEA20)	20	80	20	19.2

a: from the feed ration, *b*: calculated according to TGA measurements

FIGURES

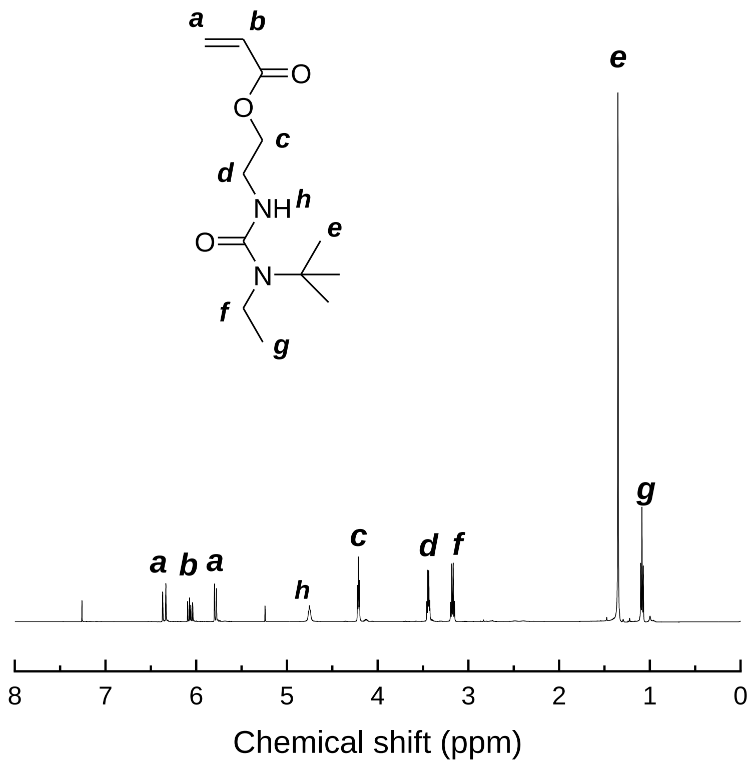

Figure S1 ¹H NMR spectrum of TEBA

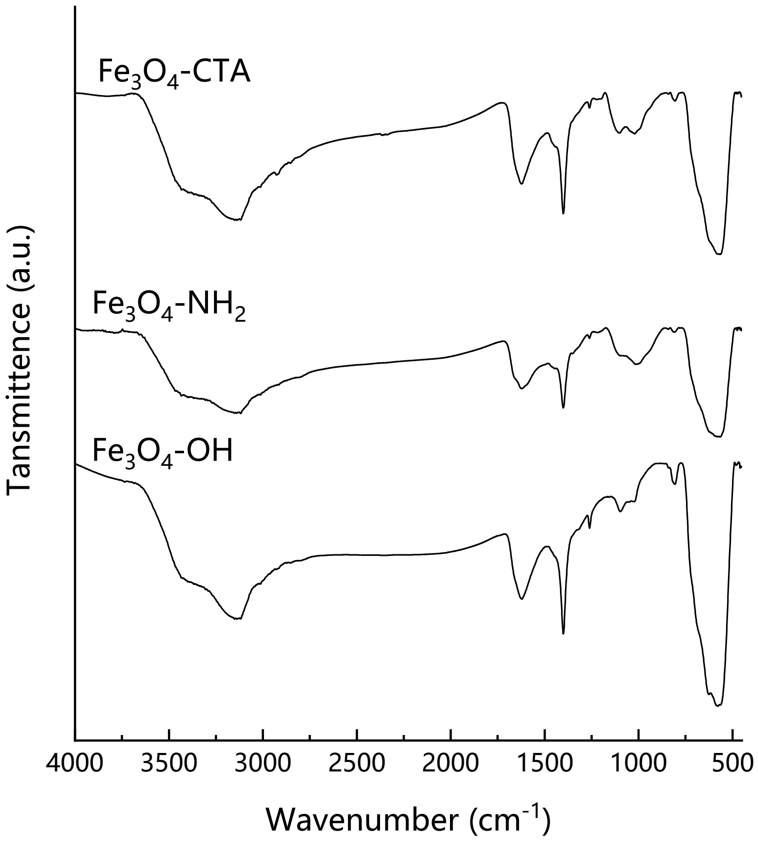

Figure S2 FTIR spectra of Fe_3O_4-OH, Fe_3O_4-NH_2 and Fe_3O_4-CTA

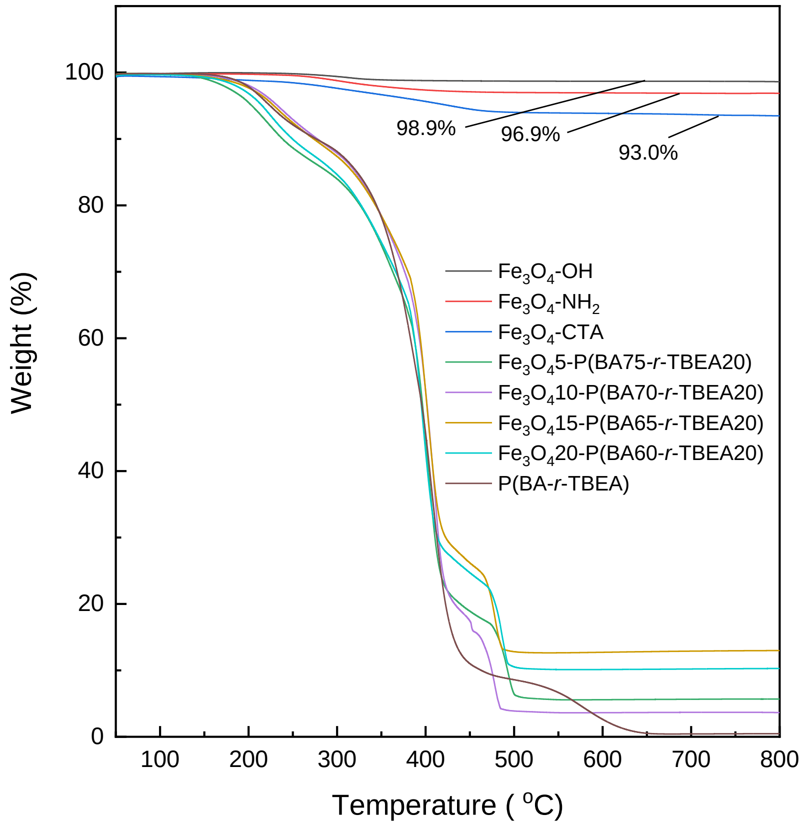

Figure S3 TGA curves of Fe_3O_4 NPs and nanocomposites

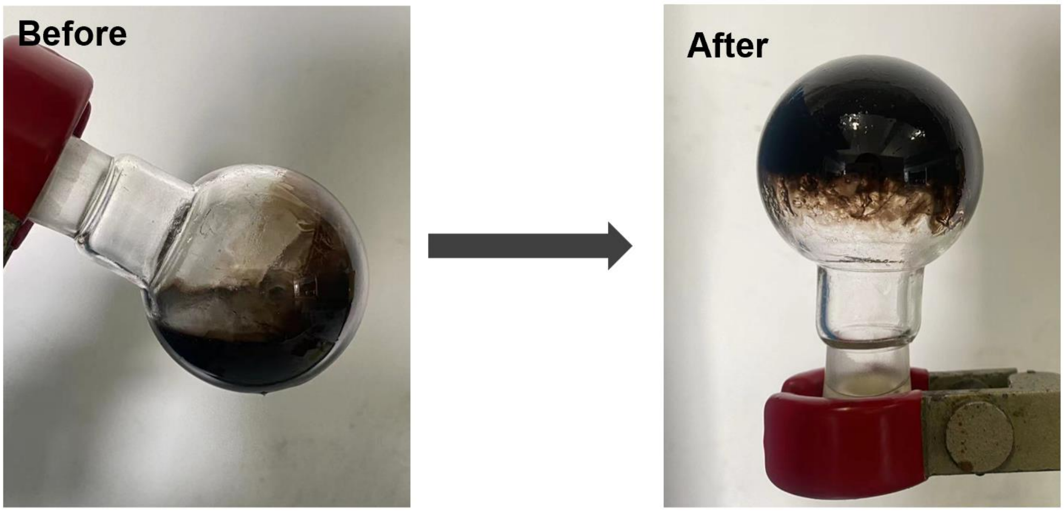

Figure S4 Photographs of the mixture of 1,2-bis(*tert*-butyl)ethylenediamine) with 10 wt% of $Fe_3O_4$15-*g*-P(BA65-*r*-TBEA20) copolymer before and after crosslinking

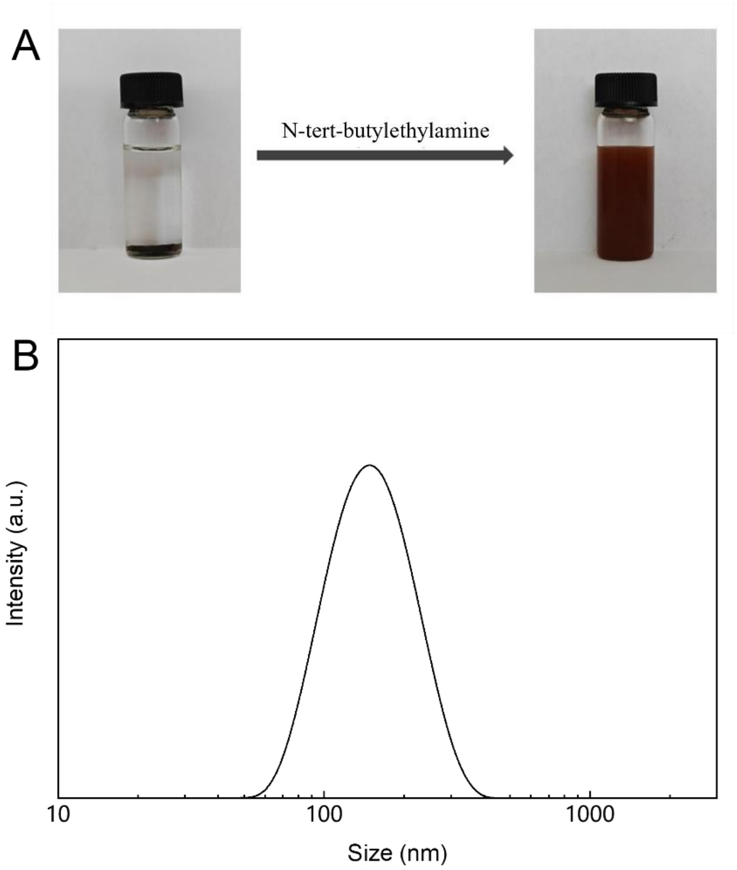

Figure S5 Photographs of solubility tests for $Fe_3O_4$15-*g*-P(BA65-*r*-TBEA20) nanocomposite: left) swollen with 1,4-dioxane; right) held at room temperature for 24 hours after N-*tert*-butylethylamine was added

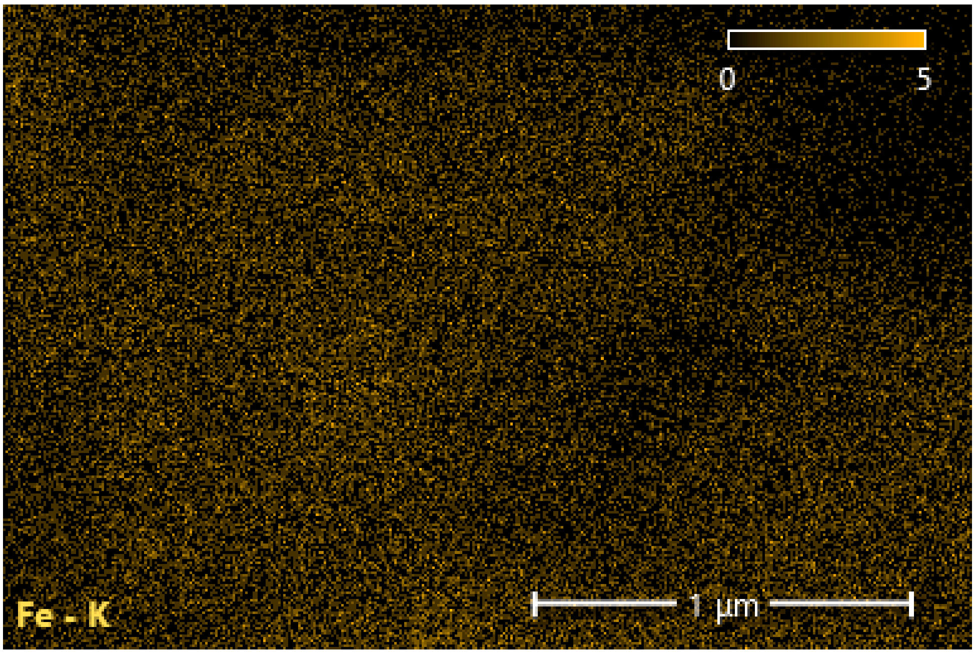

Figure S6 Iron element mapping image of $Fe_3O_4$15-*g*-P(BA65-*r*-TBEA20) nanocomposite

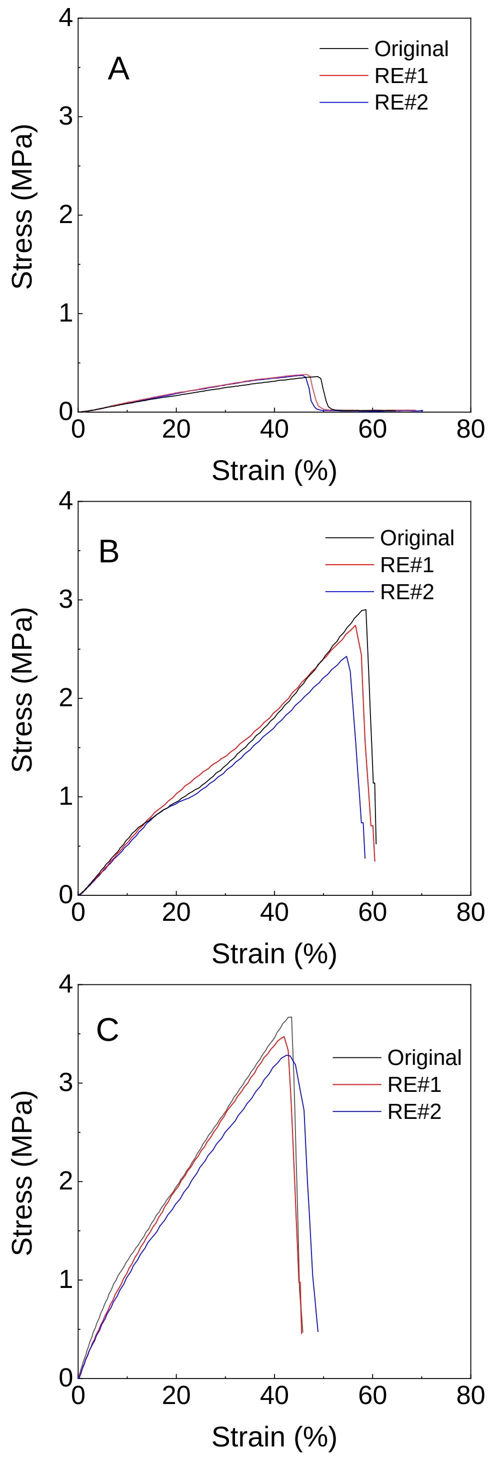

Figure S7 Stress-strain curves of the nanocomposites: (A) $Fe_3O_4$15-*g*-P(BA75-*r*-TBEA10), (B) $Fe_3O_4$15-*g*-P(BA55-*r*-TBEA30) and (C) $Fe_3O_4$15-*g*-P(BA45-*r*-TBEA40) before and after reprocessing

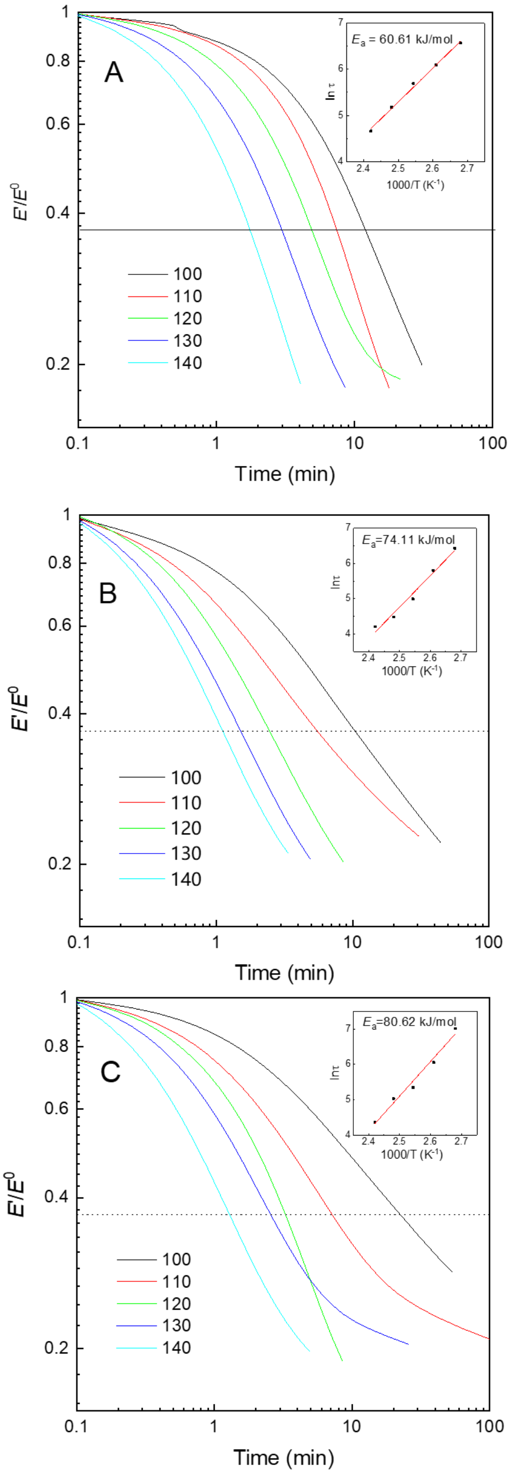

Figure S8 Stress relaxation curves of the nanocomposites: (A) $Fe_3O_4$15-*g*-P(BA75-*r*-TBEA10), (B) $Fe_3O_4$15-*g*-P(BA55-*r*-TBEA30) and (C) $Fe_3O_4$15-*g*-P(BA45-*r*-TBEA40)

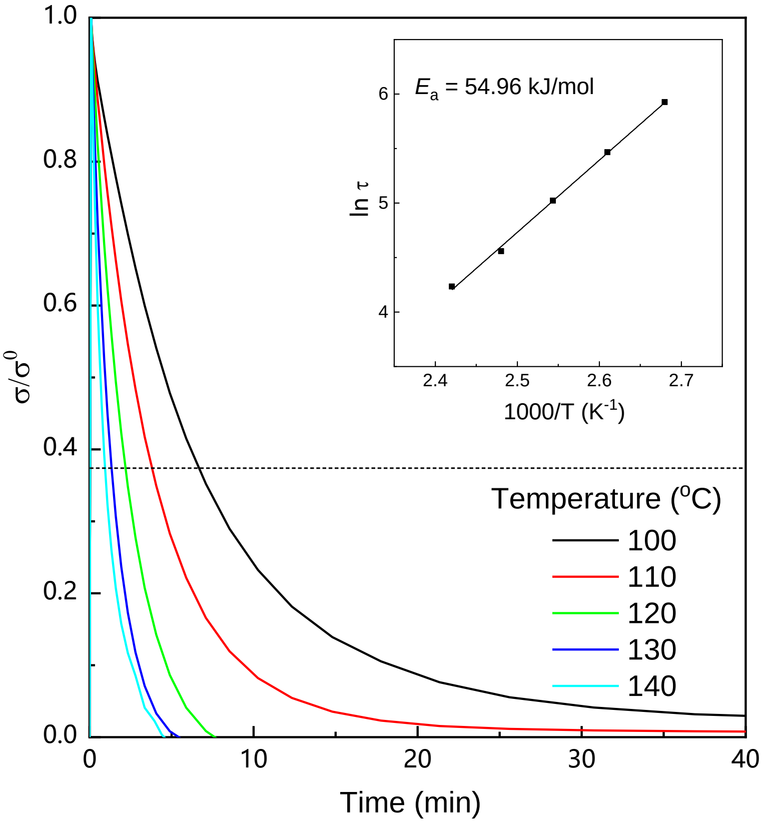

Figure S9 Stress relaxation curves of P(BA80-*r*-TBEA20) nanocomposite

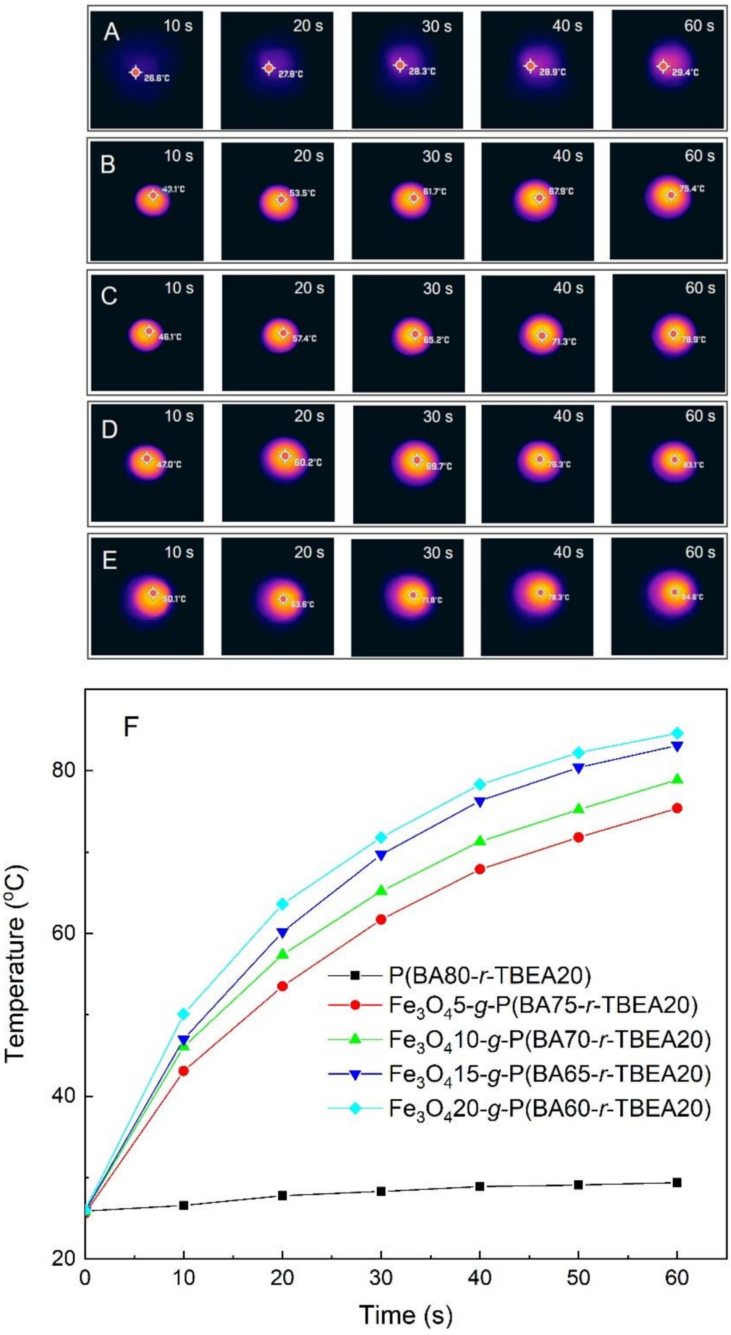

Figure S10 Surface temperature images of nanocomposites: (A) P(BA80-*r*-TBEA20), (B) $Fe_3O_4$5-*g*-P(BA75-*r*-TBEA20), (C) $Fe_3O_4$10-*g*-P(BA70-*r*-TBEA20), (D) $Fe_3O_4$15-*g*-P(BA65-*r*-TBEA20), (E) $Fe_3O_4$20-*g*-P(BA60-*r*-TBEA20), (F) surface temperature profiles of the nanocomposites as function of the time

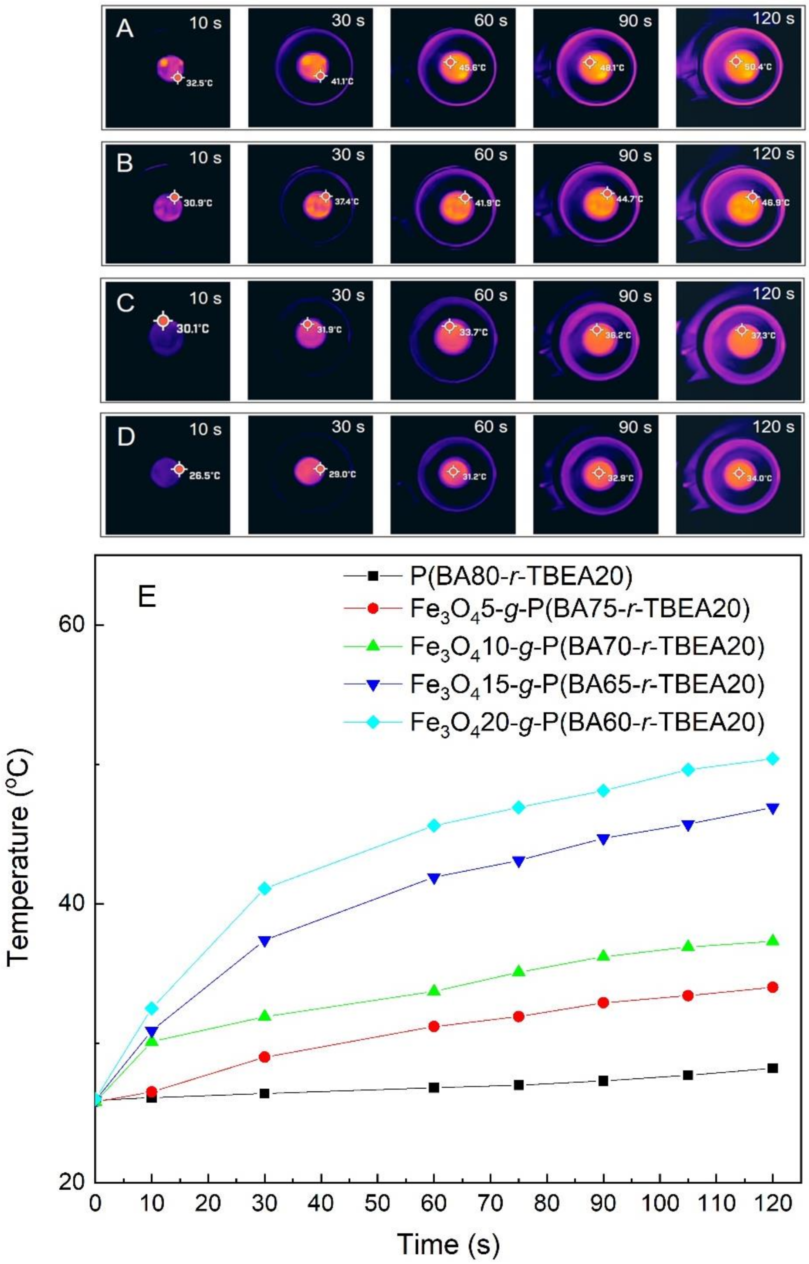

Figure S11 Surface temperature images of nanocomposites under the magnetic field: (A) $Fe_3O_4$20-*g*-P(BA60-*r*-TBEA20), (B) $Fe_3O_4$15-*g*-P(BA65-*r*-TBEA20), (C) $Fe_3O_4$10-*g*-P(BA70-*r*-TBEA20), (D) $Fe_3O_4$5-*g*-P(BA75-*r*-TBEA20), (E) surface temperature profiles of the nanocomposites as function of the time
